# Determination of 355 Pesticides in Lemon and Lemon Juice by LC-MS/MS and GC-MS/MS

**DOI:** 10.3390/foods12091812

**Published:** 2023-04-27

**Authors:** Sule Aslantas, Ozgur Golge, Miguel Ángel González-Curbelo, Bulent Kabak

**Affiliations:** 1 Department of Food Engineering, Faculty of Engineering, Hitit University, Corum 19030, Turkey; 2 Department of Gastronomy and Culinary Arts, Faulty of Tourism, Alanya Alaaddin Keykubat University, Alanya 07425, Turkey; 3 Departamento de Ciencias Básicas, Facultad de Ingeniería, Universidad EAN, Calle 79 No. 11-45, Bogotá 110221, Colombia

**Keywords:** food safety, lemon, GC-MS/MS, LC-MS/MS, method validation, QuEChERS, pesticides

## Abstract

While pesticides have become a primary tool in modern agriculture, these compounds remain a high priority on the list of consumer concerns regarding food safety. The use of pesticides in the production and post-harvesting of lemon fruits is widely used to ensure agricultural yield and fruit quality. Therefore, monitoring studies on citrus fruits to enforce regulatory compliance and ensure food safety is in great demand. The aim of this study was to monitor multi-class pesticide residues in lemon fruits commercialized in Turkey. The transmission of residues that existed on the outer surface of the fruit into its juice was also studied. Whole fruits and lemon juice samples were prepared using the quick, easy, cheap, effective, rugged and safe (QuEChERS) methodology prior to analysis. For the screening and quantification of 355 pesticide residues, liquid chromatography-tandem mass spectrometry (LC-MS/MS) and gas chromatography-tandem mass spectrometry (GC-MS/MS) were used. The analytical method has been shown to have a sufficiently low limit of quantification with respect to current maximum residue limits (MRLs) for all target analytes. The obtained recovery and precision parameters fulfilled the requirements in DG SANTE guidelines. The in-house validated analytical method was then applied for the determination of 355 pesticide substances in 100 whole fruit samples and their juices. Sixteen different residues were detected in 43% of lemon fruits, whereas 57 lemon samples were pesticide-free. The MRLs exceedances were recorded in 29 lemon samples. The most frequently detected (17%) pesticide in lemon fruits was chlorpyrifos-methyl, with a range of 0.013–0.098 mg kg^−1^. A lower frequency was detected for metamitron (10%, 0.027–0.118 mg kg^−1^), buprofezin (9%, 0.023–0.076 mg kg^−1^), pyriproxyfen (9%, 0.021–0.102 mg kg^−1^) and malathion (7%, 0.100–0.482 mg kg^−1^) in whole fruits. However, none of the pesticide residues were detected in lemon juice samples. These results showed that target analytes are unable to penetrate the lemon exocarp and/or endocarp.

## 1. Introduction

Citrus fruits, which belong to the Rutaceae family, are the most popular and widely grown fruit group in the world, with approximately 158 million metric tons per year grown [1]. The lifespan of the citrus tree, which has a perennial growth habit, is approximately 50 years. These plants mainly originate from the tropical and subtropical regions in Asia, Oceania and the Mediterranean Basin [2]. China ranks first in citrus production, with 44 million metric tons (approximately 28% of world production), followed by Brazil (19.7 million metric tons) and India (14 million metric tons). Turkey ranks 7th among citrus producing countries, with approximately 4.3 million tons of production (2.72% of world production) in 2019. Among the citrus varieties, oranges are the most cultivated and traded citrus fruits and accounted for almost half of global 2020/2021 production, followed by tangerine (22.4%), lemon (12.7%), grapefruit (5.9%) and other citrus fruits [1].

Lemons (*Citrus limon*), like other citrus fruits, are an excellent source of vitamin C and provide a multitude of other essential nutrients, such as minerals (potassium, calcium, magnesium, phosphorus and iron) and dietary fibre [3]. The lemon fruit and its juice contain high amounts of polyphenols (apigenin, hesperidin, naringin, quercetin and others) and phenolic acids (ferulic acid and synaptic acid) [4,5,6]. The juice of the fruit, valued for its tart, tangy and fresh character, is a commonly used ingredient in both home recipes and commercial ones, and is also a component of many processed foods and beverage formulas [7].

Following the growing trend of the last decade, global lemon and lime production in 2020 reached more than 21 million metric tons. India is the world’s largest producer, accounting for 17.4% of global production. Mexico and China come in second and third, with 13.5% and 12.8% of the market share, respectively. Turkey is the top-producing country in Europe, with a crop of approximately 1.2 million metric tons (5.6% of global production). In 2020, Turkey was the third largest exporter of lemons in the world after Mexico and Spain, accounting for 11.6% of the total, with 468,729 metric tons [1].

Lemon trees and lemons are vulnerable to many citrus diseases, which can be fungal, bacterial or viral, or be caused by insects (aphids and thrips). The most common diseases that threaten the lemon industry are citrus canker (*Xanthomonas* spp.), bacterial canker (*Pseudomonas syringae*), lemon scab (*Elsinoe* spp.), anthracnose (*Colletotrichum* spp.), *Armillaria* root rot, collar rot (*Phytophthora citrophthora*), *Phytophthora* root rot (*Phytophthora citrophthora*, *Phytophthora nicotianae*), blue rot (*Penicillium italicum*), green rot (*Penicillium digitatum*) and *Alternaria* rot (*Alternaria* spp.) [2,8,9].

The application of pesticides is the main method carried out to control pre-harvest and post-harvest diseases of lemon and other citrus fruits. Although pesticides provide increased productivity in agricultural production, they can cause certain acute and long-term negative health impacts and may induce toxic effects on various land and aquatic organisms in their excessive use [10,11]. To protect public health, controlling and regulating pesticide use in agricultural production and monitoring their levels in fruits and vegetables is very important. National and international organisations set maximum residue limits (MRLs) for pesticides in agricultural products to provide standards for food safety and foster international trade. In Turkey, the Ministry of Agriculture and Forestry is responsible for the determination of safe amounts of pesticide residues in animal- and plant-based foods and sets maximum residue levels (MRLs) [12] based on the European Union Regulation (EC) No 396/2005 [13]. The allowable levels of pesticides in food products depend on the type of pesticide and the food product. For example, the MRL for azoxystrobin in lemon and other citrus fruits is set at 15 mg kg^−1^, while the MRL for azoxystrobin in apples is set at 0.01 mg kg^−1^. It is important to note that MRLs are set with a significant margin of safety to ensure that the amount of pesticide residue in food products is safe for human consumption. The MRLs are based on extensive scientific research and risk assessments to ensure that they do not pose a risk to human health [14].

Fruits, including citrus fruits, are the main contributors to overall dietary exposure to pesticide residues. Several studies have shown that lemon fruits contain numerous as well as high amounts of registered or unregistered pesticide residues [15,16,17,18,19,20,21,22]. In 2021, 12% of pesticide notifications (*n* = 292) on the product category fruits and vegetables originating from Turkey were related to lemon fruits [23]. On 3 November 2021, the European Commission published a regulation by which it has been decided to increase the official control of Turkish lemons to 20% due to the emergence of health risks from pesticide residues in lemon fruits imported from Turkey, while random controls have previously been carried out with 10% frequency [24].

The aim of this study was to monitor pesticide residues in Turkish lemons using the quick, easy cheap, effective, rugged and safe (QuEChERS) sample preparation approach combined with liquid chromatography-tandem mass spectrometry (LC-MS/MS) and gas chromatography-tandem mass spectrometry (GC-MS/MS). The transmission of pesticides that existed on the outer surface of the lemon into its juice was also studied.

## 2. Materials and Methods

### 2.1. Standards, Chemicals and Reagents

Standards of 355 pesticides (309 LC-amenable and 46 GC-amenable) with >95% purity were from four suppliers: A2S Analytical Standard Solutions Co. (Saint Jean d’Illac, France), ChemService (West Chester, PA, USA), Dr. Ehrenstorfer GmbH (Augsburg, Germany) and Sigma–Aldrich (Steinheim, Germany). The selected pesticides included the main pesticides used by the citrus farmers and other registered, non-registered and banned active substances in the cultivation of citrus fruits in Turkey. Two mix stock solutions of multi-residue pesticides (mix 1: 309 pesticides, mix 2: 46 pesticides) were prepared at 10 mg L^−1^ in acetonitrile. The multi-residue stock standard solutions were stored at −18 °C in glass vials for a maximum of six months. From these stock solutions, mixed standard working solutions were prepared in acetonitrile and used in the preparation of matrix-matched calibration standards and method recovery studies.

Acetic acid (glacial acetic acid, CH_3_COOH, reagent grade, 100%), acetonitrile (ACN, LC-MS grade), methanol (MeOH, LC-MS grade) and ammonium formate (HCOONH_4_, analytical grade, ≥99% purity) were purchased from Sigma–Aldrich (Steinheim, Germany). Formic acid (CH_2_O_2_, LC-MS grade, 98% purity), anhydrous magnesium sulphate (MgSO_4_, analytical grade) and anhydrous sodium acetate (NaOAc, analytical grade) were obtained from Merck (Darmstadt, Germany). Primary secondary amine (PSA, 40 µm particle size) was from Supelco (Bellefonte, PA, USA). Ultrapure water (18.2 MΩ cm) was obtained using a Milli-Q3 system from Millipore (Molsheim, France).

### 2.2. Samples

The lemon samples (*n* = 100) were collected during the 2020/2021 growing season from supermarkets, groceries and bazaars in Corum province, Turkey. The sample sizes were at least 500 g and were taken during the period of November 2020–January 2021. Each randomly selected unwashed lemon fruit was cut in half across the width of the lemon with a sharp knife, one half of which was squeezed into a bowl to obtain lemon juice, and the other half with the peel was homogenized by a home food processor (Fakir, Stuttgart, Germany) to obtain small and uniform particle sizes. Both fruit (*n* = 100) and lemon juice (*n* = 100) samples were stored under cool conditions (4–8 °C) until subjection to the sample extraction step within 24 h.

### 2.3. Sample Preparation

**Figure 1:**
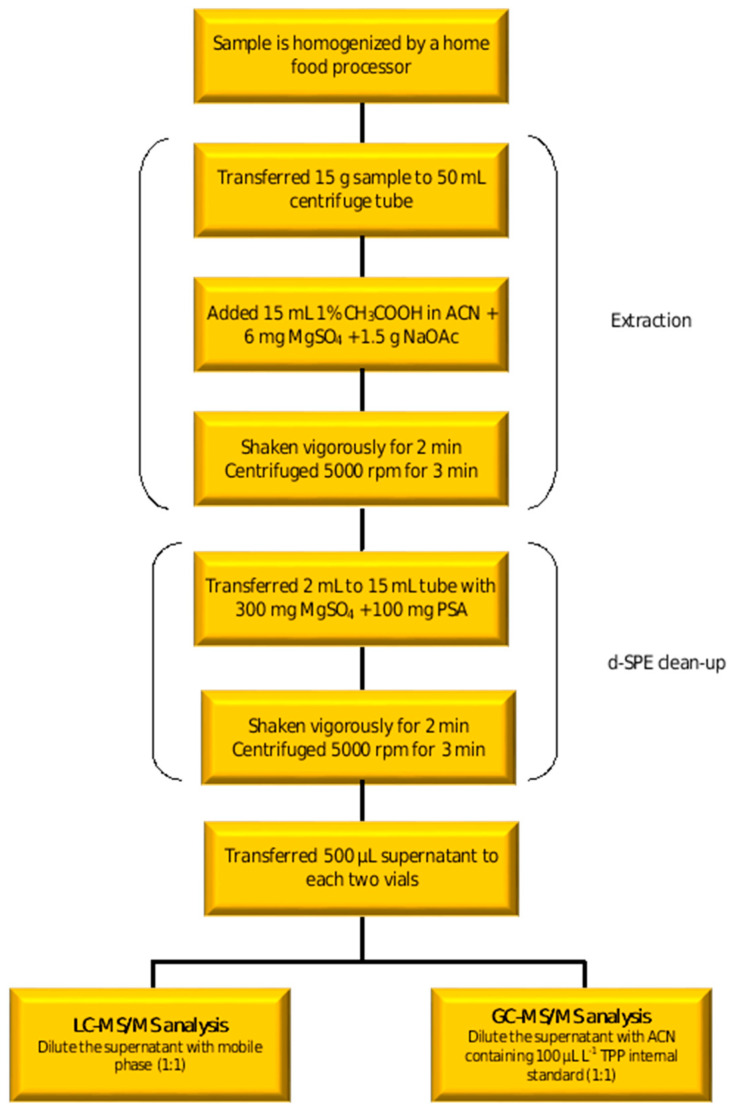
Sample preparation protocol of the QuEChERS method.

Lemon fruit with peel and fresh lemon juice matrices were prepared for analysis using a modified QuEChERS approach [25] (Figure 1), which involves two steps: extraction and dispersive solid phase extraction (d-SPE) clean-up. For extraction, 15 g of homogenized sample, 15 mL of acetonitrile acidified with 1% acetic acid and 7.5 g of MgSO_4_/NaOAc (4:1, *w*/*w*) as phase partition salts were used. In the d-SPE clean-up step, 150 mg of MgSO_4_ to remove water and 50 mg of PSA to remove carbohydrates, organic acids, fatty acids and pigments were used per millilitre of supernatant. 

### 2.4. LC-MS/MS Analysis

A ThermoFisher Scientific UltiMate 3000 HPLC system (Bremen, Germany) was used for the separation of 309 LC-amenable analytes in lemon fruit and fresh lemon juice samples. The system was equipped with a binary pump, an autosampler, column oven and degasser. The instrument was controlled by the Thermo Scientific Xcalibur v.4.0 software. Separation of multi-residue analytes was achieved using an Accucore Vanquish C-18 reversed-phase column (2.1 × 100 mm, 1.5 µm particle size, ThermoFisher Scientific) at 40 °C. The chromatographic separation was performed in gradient mode, as described in Table 1, using water–methanol (98:2, *v*/*v*) (mobile phase A) and methanol–water (98:2, *v*/*v*) (mobile phase B), both of which containing 0.1% formic acid and 5 mM ammonium formate. An injection volume of 10 μL was applied onto the column.

The mass spectrometry system employed in this study consisted of a ThermoFisher Scientific Q-Exactive Focus Orbitrap MS (Bremen, Germany) equipped with an electrospray ionisation (ESI) interface operating in positive ionisation mode with a spray voltage of 2800 V. The spray and vaporizer temperatures were set at 320 and 295 °C, respectively, while the spray voltage was maintained at 2.8 kV. Data processing for full scan was conducted using TraceFinder™ software, while multiple reaction monitoring (MRM) data were acquired and processed for all compounds. Identification of target pesticide residues was performed in accordance with the SANTE/11312/2021 guidelines [26], using two specific MRM transitions (Table S1) for each residue. Quantification was performed by selecting the transition with the most abundant product transition ion in MRM mode.

### 2.5. GC-MS/MS Analysis

The lemon and lemon juice samples treated with the QuEChERS approach were analysed in multiple reaction monitoring (MRM) mode with a TSQ9000 GC-MS/MS system (ThermoFisher Scientific, Bremen, Germany) for the determination of 46 GC-amenable residues. Data were acquired using the TraceFinder™ software. The chromatographic and mass spectrometer parameters are summarized in Table 2. The optimized MRM parameters are presented in Table S2.

### 2.6. Method Validation

The scope of this in-house validation was to verify that the results obtained by applying this method fulfil the requirements set by SANTE/11312/2021 guidelines [26]. The performance of the analytical method was evaluated by checking the specificity, linearity, limit of quantification (LOQ), recovery, precision and measurement uncertainty. The specificity was confirmed based on the presence of accurate parent masses and fragment ions at the correct retention time (±0.1%), corresponding to the matrix-matched pesticide standards. To assess the linearity of the method, blank lemon matrices, created by the combining and mixing of five homogenized lemon blank samples, were spiked with target analytes at seven concentrations of 0.0025, 0.05, 0.01, 0.02, 0.04, 0.08 and 0.16 mg kg^−1^. The LOQ, recovery and precision were evaluated based on data from recovery experiments. The LOQ was determined in accordance with the SANTE guidelines by calculating the standard deviation (SD) of a blank lemon matrix spiked with pesticide mix at the lowest spiking level of 0.01 mg kg^−1^, ensuring acceptable accuracy (70–120%) and precision (relative standard deviations (RSD) ≤ 20%). The method recovery and precision were evaluated by recovery studies in which blank whole fruit samples were spiked with pesticide mix at two concentration levels of 0.01 and 0.05 mg kg^−1^. Six replicates were prepared for each set of experiments. The spiked materials were then analysed according to the method protocol, and target analytes were quantified using the obtained matrix-matched calibration curves. The recovery of target analytes was calculated as follows (Equation (1)):

(1) $$\%\;recovery=\frac{\mathrm{measured}\;\mathrm{concentration}}{\mathrm{spiked}\;\left(\mathrm{added}\right)\;\mathrm{concentration}}\times 100\;\;\;\;\;\;$$

The repeatability (*n* = 6) and within-laboratory reproducibility (*n* = 12) were calculated as RSD of replicate measurements in which the experiments were carried out on the same day by one operator and on three consecutive days by two different operators, respectively. Based on EURACHEM guidelines [27], the expanded measurement uncertainty (U) was determined through the multiplication of a coverage factor of *k* = 2, which corresponds to a confidence level of approximately 95%, with the combined standard uncertainty ($u_{c}$). The combined standard uncertainty was calculated by considering the uncertainty associated with within-laboratory reproducibility ($u\left(RSD_{wR}\right)$) and the method trueness (bias) ($u\left(bias\right)$), as expressed in the following equation (Equation (2)):

(2) $$u_{c}=\sqrt{u{\left(RSD_{wR}\right)}^{2}+u{\left(bias\right)}^{2}\;}\;\;\;\;\;\;\;\;\;\;\;\;\;$$

## 3. Results

### 3.1. Method Validation Data

**Figure 2:**
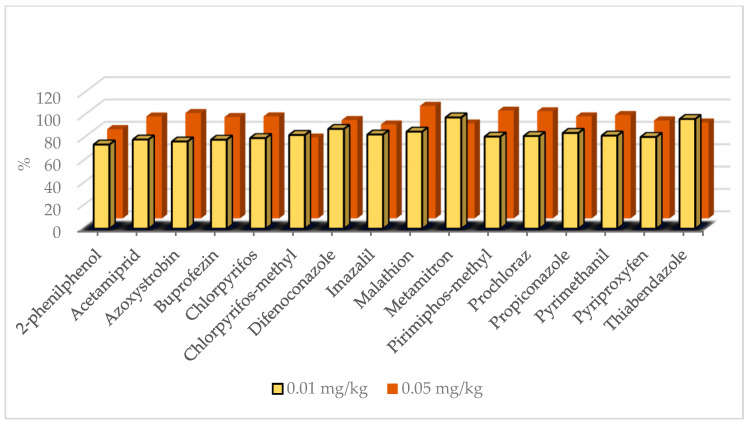
Recoveries of detected pesticides in whole lemon fruit matrix.

**Figure 3:**
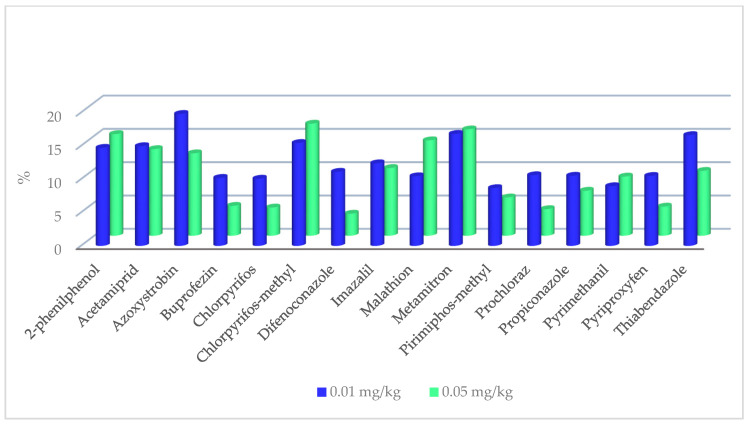
The relative standard deviations (RSDs) of detected residues in whole lemon matrix under reproducibility conditions.

The method validation data for the whole lemon matrix across the 309 LC-amenable and 46 GC-amenable pesticides are summarized in Tables S1 and S2, respectively. Calibration was assessed in the lemon matrix and was found to be acceptable for all compounds to SANTE/11312/2021 guidelines. The response was linear for target analytes over a range of 0.0025 to 0.16 mg kg^−1^ (*R*^2^ > 0.991, residuals < 20%). For all pesticides, except for pyrimidifen, the LOQs were at or lower than 0.01 mg kg^−1^. Recoveries obtained for the pesticides at two spiking levels in the whole lemon matrix were within the acceptable tolerance of 70% to 120% range. Recoveries for LC-amenable and GC-amenable pesticides varied from 72.7% to 115.3% and from 71.8% to 116.1%, respectively. Recoveries of detected pesticides at two spiking levels are illustrated in Figure 2. Repeatability (*n* = 6) and reproducibility (*n* = 12) of the analytical method were determined by injecting two spiking levels from the matrix-matched curves. The RSDs under repeatability and reproducibility conditions were within the range of 3.25–17.98% and 3.05–19.98% for LC-amenable pesticides and 1.03–15.19% and 6.78–19.79% for GC-amenable pesticides, respectively. The RSDs under reproducibility conditions for detected pesticides at two spiking levels are shown in Figure 3. The expanded measurement uncertainty, which encompassed trueness and reproducibility, was lower than 50% for all pesticides, as specified by SANTE/11312/2021 guidelines.

### 3.2. Pesticide Residues in Whole Lemon Fruit and Lemon Juice

**Figure 4:**
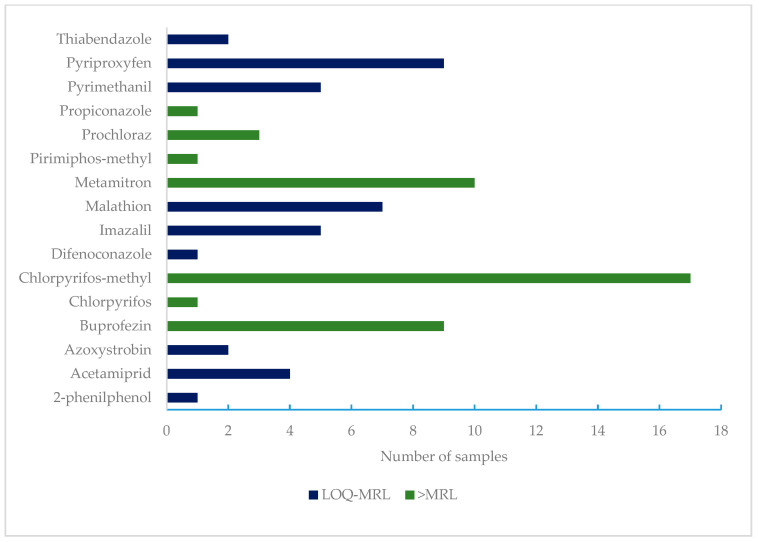
The frequency distribution of pesticide residues in whole lemon fruit samples.

During the 2020/2021 growing season, a total of 100 lemon samples consumed in Turkey were monitored for 355 pesticides; 57% of the samples were free from pesticide residues. While 14 samples of lemon fruits contained one or several detectable residues within the legally permitted concentrations, European Union (EU) MRL exceedances were identified in 29 samples. Of the 355 pesticides analysed in lemon fruits, 16 different pesticides were found in concentrations above the LOQ: 2-phenylphenol, acetamiprid, azoxystrobin, buprofezin, chlorpyrifos, chlorpyrifos-methyl, difenoconazole, imazalil, malathion, metamitron, pyrimethanil, prochloraz, propiconazole, pyriproxyfen, pirimiphos-methyl and thiabendazole. Among the 16 pesticides, 13 of them are currently approved in the EU, whereas three pesticides, chlorpyrifos, chlorpyrifos-methyl and propiconazole, are non-approved for use. The frequency distribution of pesticide residues in lemon fruits is shown in Figure 4.

In 22 lemon samples, only one residue was quantified. Multiple residues (more than one residue) were detected in 21 lemon samples, of which 11 samples had two residues, seven samples had three residues, two samples had four residues and only one sample had five residues. The residues detected in lemon fruits belonged to the 11 different chemical classes of pesticides. Among the chemical class of pesticides detected in lemon fruits, the most common was organophosphates. However, the chemical class of imidazole (imazalil and prochloraz) had the highest mean concentration (1.305 mg kg^−1^) in lemon fruits, followed by phenolic compound (2-phenylphenol), with a concentration of 0.552 mg kg^−1^, and organophosphate compounds (chlorpyrifos, chlorpyrifos-methyl, malathion and pirimiphos-methyl), with 0.476 mg kg^−1^. The range of measured pesticide values and frequency of detection in lemon fruits are presented in Table S3.

Among the residues, chlorpyrifos-methyl was the most frequently detected analyte in the lemon fruit samples, with a frequency of 17%. The concentration of chlorpyrifos-methyl in lemon samples ranged between 0.013 and 0.098 mg kg^−1^ (mean = 0.056 mg kg^−1^), and they were all higher than the EU MRL of 0.01 mg kg^−1^. Moreover, one lemon sample contained chlorpyrifos at a concentration of 0.073 mg kg^−1^. Chlorpyrifos and chlorpyrifos-methyl are broad-spectrum organophosphate insecticides and acaricides which inhibit acetylcholinesterase, producing neurotoxic effects in insects and non-target organisms [28]. Both active substances are used in a wide range of products including fruits, especially citrus fruits and vegetables, as well as in viticulture and turf grasses. Due to their genotoxic potential and epidemiological evidence of developmental neurotoxicity in children, both agents have been banned in the European Union, the United States and Turkey since the beginning of 2020. Within the last two decades, the acceptable daily intake (ADI) established in 2006, initially set at 0.01 mg kg^−1^ body weight (b.w.) per day, has undergone several reductions in response to assessments conducted by the European Food Safety Authority (EFSA). These reductions have resulted in a decrease in authorized usage and MRLs for chlorpyrifos, ultimately leading to a gradual reduction in estimated exposure levels for European citizens. In 2012, new toxicological studies necessitated a review, prompting EFSA to propose a reduction in the ADI to 0.001 mg/kg body weight per day [29]. The EFSA’s 2019 conclusion stated that toxicological reference values could not be established for both chlorpyrifos [30] and chlorpyrifos-methyl [31], because of their unclear genotoxicity.

Metamitron was the second most frequently found compound in lemon fruits. It was recorded in 10 samples at levels ranging from 0.027 to 0.118 mg kg^−1^, with a mean level of 0.073 mg kg^−1^. The MRL of 0.01 mg kg^−1^ for metamitron was exceeded for all positive samples. Metamitron is an active ingredient that inhibits photosynthesis by disrupting the photosynthetic apparatus. Specifically, it temporarily inhibits the transfer of electrons between the primary and secondary quinones of photosystem II. In recent years, metamitron has been used as a chemical fruit-thinning agent for several fruits, including apple, pear and citrus fruits [32].

The approved insecticide buprofezin was measured in nine lemon samples in concentrations of 0.023–0.076 mg kg^−1^ (mean = 0.050 mg kg^−1^), which were all higher than EU MRL of 0.01 mg kg^−1^. A similar frequency was observed for pyriproxyfen insecticide within the legal limit of 0.6 mg kg^−1^. The concentration of pyriproxyfen varied from 0.021 to 0.102 mg kg^−1^ (mean = 0.061 mg kg^−1^). The fungicide malathion was measured in seven samples (0.100–0.482 mg kg^−1^, mean = 0.291 mg kg^−1^), but all were below the EU MRL of 2 mg kg^−1^. Other fungicides, imazalil and pyrimethanil, were measured in five samples in concentrations of 0.419–1.172 mg kg^−1^ (mean = 0.796 mg kg^−1^) and 0.033–0.548 mg kg^−1^ (0.290 mg kg^−1^), respectively, which were far below their EU MRLs. Acetamiprid insecticide was detected in four lemon samples at levels varying from 0.051 to 0.128 mg kg^−1^ (mean = 0.090 mg kg^−1^). In addition, four fungicides, azoxystrobin (two samples, 0.040–0.044 mg kg^−1^), thiabendazole (two samples, 0.104–0.111 mg kg^−1^), 2-phenylphenol (one sample, 0.552 mg kg^−1^) and difenoconazole (one sample, 0.025 mg kg^−1^), were detected sporadically.

The relatively high incidence of chlorpyrifos-methyl in the lemon samples showed that these active substances continued to be placed in the Turkish market for use by citrus farmers. This result confirms our previous study, in which chlorpyrifos was the predominant pesticide in Turkish oranges at levels ranging from 0.01 to 0.09 mg kg^−1^ [33]. In a previous report by Azar and Kıvan [15], the frequency of pesticide residues in lemon fruits consumed in the Bursa region, Turkey, was almost twice-fold higher (83%) when compared to our results. They detected eight different pesticides, chlorpyrifos, buprofezin, carbofuran, methidathion, bromopropylate, parathion-methyl, cypermethrin and dicofol, in lemon fruit samples. It has also been reported that chlorpyrifos was the predominant residue in lemon samples. In another study by Bakırcı et al. [17], the most frequently detected pesticide in Turkish lemons marketed in the Aegean region of Turkey was chlorpyrifos (0.01–0.016 mg kg^−1^), with a frequency level of 33.3%. Other pesticides detected in lemon samples were imazalil (17.9%, 0.241–6.79 mg kg^−1^), thiabendazole (17.9%, 0.05–2.82 mg kg^−1^), bromopropylate (15.4%, 0.047–4.01 mg kg^−1^), carbendazim (15.4%, 0.014–0.334 mg kg^−1^), pyridaben (15.4%, 0.012–0.019 mg kg^−1^), pyriproxyfen (15.4%, 0.12–0.95 mg kg^−1^), tebuconazole (15.4%, 0.12–0.95 mg kg^−1^), pyrimethanil (7.7%, 0.027–0.45 mg kg^−1^), hexythiazox (5.1%, 0.01–0.014 mg kg^−1^), 2,4-D (2.6%, 0.05 mg kg^−1^) and acetamiprid (2.6%, 0.05 mg kg^−1^). In a similar study, 54 citrus fruits (23 lemons, 20 oranges, 10 tangerines and only one grapefruit sample) were sampled in twenty different orchards from the Muğla region of Turkey and monitored for 198 insecticides by LC-MS/MS and GC-MS/MS. The predominant insecticides in citrus fruits were pyriproxyfen (64.8%), pyridaben (51.9%) and chlorpyrifos (35.2%), with levels up to 0.091, 0.025 and 0.077 mg kg^−1^, respectively [18].

The results obtained in the present study were also confirmed by the Rapid Alert System for Food and Feed (RASFF) database. In 2022, a total of 71 notifications on pesticide residues were published for lemon fruits. All of these notifications on pesticides concerned lemon fruits originating from Turkey. Chlorpyrifos-methyl was the first source of these notifications (30 notifications, 42.3%) on lemon fruits, followed by prochloraz (16 notifications, 22.5%), chlorpyrifos (13 notifications, 18.3%), fenbutatin oxide (6 notifications, 8.5%) and buprofezin (4 notifications, 5.6%). The notifications were notified mainly by Bulgaria (*n* = 54, 76%). The remaining notifications were notified by ten countries: Poland (*n* = 3), Romania (*n* = 3), Greece (*n* = 2), Italy (*n* = 2), Slovakia (*n* = 2), Austria (*n* = 1), Croatia (*n* = 1), Hungary (*n* = 1), Latwia (*n* = 1) and Sweeden (*n* = 1) [23].

There are also several reports worldwide concerning the monitoring of residues in lemon fruits. In a Swiss study on citrus fruits, including 133 lemon samples mainly imported from Spain, 38 different residues were identified in 95% of the 164 samples. Among the residues, imazalil and thiabendazole were the most common pesticides detected in citrus fruits [34]. More recently, Calvaruso et al. [35] reported that only fenhexamid was identified among the 165 pesticides in 12 out of 20 lemon samples from the Sicily market, Italy, up to a level of 0.066 mg kg^−1^.

In a Danish monitoring program during the years 2004–2011, 370 out of 381 lemon samples (97%), mainly exported from Spain, contained 39 different residues. Imazalil and chlorpyrifos were the most frequently detected residues, and 79% of the lemon samples contained multiple residues. The MRLs were exceeded for 1% of the lemon samples [36]. In a 2019 EU report on pesticide residues in food, the occurrence data for a total of 1863 lemon samples provided from European countries were reported. 16% of lemon samples contained only one residue, whereas multiple residues were recorded in 1185 samples (64%), of which 18.7% contained two residues, 16% contained three residues, 14.2% contained four residues, 8.2% contained five residues and 6.5% contained more than five residues. In lemon samples, the MRL exceedances were observed for 6% (112 samples) of the samples; 8% of the 988 lemon samples imported from Turkey were also reported to contain non-approved pesticides [37].

The fresh lemon juice samples (*n* = 100) obtained from whole fruits were also checked in order to determine the transition of target analytes from whole fruits to lemon juice. None of the residues of fungicides, insecticides/acaricides and herbicides were detected in lemon juice samples. These results indicated that 16 pesticides did not transfer from the outer surface of the fruit to the juice inside. This suggests that the peeling of the fruits may be an effective way to remove any potential pesticide residues from the surface of the fruit. It is worth noting that this study only concentrated on a specific set of pesticides and may not be representative of all pesticide residues that could potentially be present on fruit surfaces. Additionally, the effectiveness of peeling or washing in removing pesticide residues may depend on the type of pesticide and the washing method used. This result confirms the previous observation by Omeroğlu et al. [38], who reported the residues abamectin and etoxazole were not detected in fresh orange juice obtained from contaminated oranges. However, the concentrations of buprofezin, imazalil and thiophanate-methyl decreased by 93%, 79% and 63%. In a similar report by Li et al. [39], the concentration of abamectin, cypermethrin, imidacloprid and prochloraz decreased by 46%, 94.7%, 46.5% and 81%, respectively, after processing orange fruits into orange juice.

Food processing techniques, such as the washing, peeling, juicing, cooking, canning or fermentation process, can lead to large reductions in residue levels [40]. The peeling of fresh fruits such as citrus fruits, kiwi fruits, bananas, avocado, mango and pineapple could achieve complete removal of residues from the fruit. The insecticide pirimiphos-methyl was detected in the lemon skin at concentrations of 0.5–5 mg kg^−1^ after 21–28 days of treatment in the field, whereas lemon pulp was free of measurable concentrations [41]. More recently, Kowalska et al. [42] demonstrated a considerable accumulation of pesticides in the peels of citrus fruits. Conversely, negligible amounts of residues of active substances were detected in the flesh of citrus fruits, with no recorded instances surpassing the established MRL values. In another study, the residues cypermethrin (0.6 mg kg^−1^), dimethoate (0.45 mg kg^−1^), fenthion (0.40 mg kg^−1^) and fenvalerate (0.68 mg kg^−1^) were removed completely from mangoes by peeling process [43].

## 4. Conclusions

The analytical method based on QuEChERS sample preparation followed by LC-MS/MS or GC-MS/MS determination is fit-for-purpose with LOQs ≤ 0.01 mg kg^−1^ for all target residues, except for pyrimidifen, allowing their quantification at and below the EU MRLs. The validation parameters showed that the method enables the measurement of multi-class pesticide residues in lemon fruits. In 57% of lemon fruit samples, no quantifiable residues were recorded. However, 16 different residues were found in 43 samples, 21 of which contained multiple residues. Non-approved chlorpyrifos-methyl was the predominant residue detected in whole lemon fruits. Chlorpyrifos-methyl amounts > 0.01 mg kg^−1^ were detected in 17 samples, with quantities up to 0.098 mg kg^−1^. Because chlorpyrifos-methyl is a fast-acting, effective insecticide, it was widely used in citrus fruits over a long period of time in Turkey. The high incidence of chlorpyrifos-methyl in lemon fruits consumed in Turkey confirms its ubiquitous presence in RASFF notifications notified by European countries. This study also showed that the processing of lemon fruits into their juice removed chlorpyrifos-methyl and another 15 residues detected in lemon fruits completely. This could be due to the fact that chlorpyrifos-methyl and other residues are unable to penetrate lemon exocarp (flavedo) and/or endocarp (albedo).

## Figures and Tables

**Table 1:** Gradient conditions for LC.

Time (min)	Mobile Phase A% ^a^	Mobile Phase B% ^b^	Flow (mL min^−1^)
0.0	100	0	0.3
0.5	100	0	0.3
7.0	30	70	0.3
9.0	0	100	0.3
12.0	0	100	0.3
12.1	100	0	0.3
18.0	100	0	0.3

^a^ Water–methanol (98:2, *v*/*v*) with 0.1% formic acid and 5 mM ammonium formate. ^b^ Methanol–water (98:2, *v*/*v*) with 0.1% formic acid and 5 mM ammonium formate.

**Table 2:** Chromatographic and mass spectrometry conditions.

Parameter	Chromatographic Conditions		
Column	TraceGold^TM^ TG-5MS (30 m × 0.25 mm × 0.25 µm, ThermoFisher Scientific)		
Injection mode	Splitless		
Splitless time	1.5 min		
Injection volume	1 µL		
Carrier gas (purity)	Helium (99.999%)		
Column temperature program	Rate (°C/min)	Temperature (°C)	Hold time (min)
		40	1.5
	25	90	1.5
	25	180	0
	5	280	0
	10	300	4
**Parameters**	**MS conditions**		
MS transfer line temperature	280 °C		
Ion source temperature	280 °C		
Ionisation type	Electron ionisation (EI)		
Measurement mode	Multiple reaction monitoring (MRM)		
Collision gas and pressure (psi)	Argon at 60		

## Data Availability

Data are contained within the article.

## Supplementary Materials

The following supporting information can be downloaded at: https://www.mdpi.com/article/10.3390/foods12091812/s1, Table S1: MRM transitions and in-house validation data for 309 pesticides in whole lemon fruit by LC-MS/MS; Table S2: MRM transitions and in-house validation data for 46 pesticides in whole lemon fruit by GC-MS/MS; Table S3: The range of measured pesticide values and frequency of detection in lemon fruits.
