# Supplementary material for: Determination of 355 Pesticides in Lemon and Lemon Juice by LC-MS/MS and GC-MS/MS
Source: Foods. 2023 Apr 27;12(9):1812. doi: 10.3390/foods12091812 (PMC10178108; doi:10.3390/foods12091812)
Supplement: Supplementary file 1 [file foods-12-01812-s001.zip › foods-2323967-supplementary.pdf]

**Supplementary material**

**Table S1. MRM transitions and in-house validation data for 309 pesticides by LC-MS/MS.**

| Pesticide | MRM transitions, *m/z* (collision energy, eV) | | LOQ (mg kg$^{-1}$) | Recovery (%) | | Repeatability (%RSD, *n*=6) | | Within-laboratory reproducibility (%RSD, *n*=12) | | *U* (%) |
|---|---|---|---|---|---|---|---|---|---|---|
| | quantification | qualification | | 0.01 mg kg$^{-1}$ | 0.05 mg kg$^{-1}$ | 0.01 mg kg$^{-1}$ | 0.05 mg kg$^{-1}$ | 0.01 mg kg$^{-1}$ | 0.05 mg kg$^{-1}$ | |
| 2.4-D | 219.0/160.8 (20) | 219.0/124.9 (38) | 0.007 | 81.55 | 73.16 | 7.99 | 3.09 | 14.83 | 15.04 | 23 |
| 2-phenylphenol | 170.1/155.0 (22) | 170.1/141.0 (34) | 0.009 | 75.06 | 79.80 | 3.25 | 7.87 | 14.72 | 15.26 | 29 |
| Acephate | 184.0/142.9 (13) | 184.0/124.9 (25) | 0.008 | 82.02 | 86.72 | 10.13 | 9.87 | 11.90 | 11.36 | 25 |
| Acetamiprid | 223.1/126 (31) | 223.1/99.0 (55) | 0.002 | 79.52 | 91.11 | 10.39 | 2.81 | 14.97 | 13.01 | 17 |
| Acibenzolar-s-methyl | 211.0/136.1 (41) | 211.0/91.1 (29) | 0.008 | 85.64 | 86.75 | 14.68 | 4.59 | 17.11 | 18.38 | 26 |
| Aldicarb | 208.1/116.0 (11) | 208.1/88.9 (20) | 0.007 | 77.63 | 89.49 | 10.76 | 3.41 | 13.74 | 15.42 | 28 |
| Aldicarb-sulfone | 223.2/86.1 (21) | 223.2/147.9 (13) | 0.006 | 76.67 | 98.07 | 11.79 | 7.22 | 11.42 | 11.79 | 19 |
| Aldicarb-sulfoxide | 207.2/132.0 (11) | 207.2/88.9 (19) | 0.006 | 82.78 | 81.89 | 8.07 | 6.59 | 10.25 | 11.04 | 27 |
| Allidochlor | 174.0/80.9 (8) | 174.0/97.9 (24) | 0.004 | 80.20 | 89.45 | 9.44 | 3.60 | 8.29 | 5.04 | 19 |
| Aminocarb | 209.1/152.1 (19) | 209.1/137.2 (31) | 0.007 | 77.11 | 85.68 | 6.20 | 4.16 | 13.19 | 8.36 | 32 |
| Amitraz | 294.0/163.0 (17) | 294.0/122.0 (41) | 0.003 | 83.99 | 86.03 | 14.81 | 8.55 | 15.60 | 13.38 | 35 |
| Anilofos | 368.0/199.0 (21) | 368.0/125.0 (45) | 0.003 | 82.94 | 98.44 | 8.31 | 2.57 | 8.79 | 14.85 | 19 |
| Atrazine-desisopropyl | 174.1/104.1 (33) | 174.1/132.1 (25) | 0.009 | 84.81 | 91.60 | 5.69 | 7.40 | 8.12 | 8.55 | 31 |
| Atrazine-desethyl | 188.1/146.1 (23) | 188.1/110.1 (31) | 0.003 | 74.71 | 87.33 | 9.18 | 1.91 | 11.39 | 10.29 | 19 |
| Azaconazole | 300.0/159.0 (37) | 300.0/231.0 (23) | 0.005 | 77.97 | 92.94 | 11.06 | 3.90 | 15.60 | 15.21 | 21 |
| Azamethiphos | 325.0/183.0 (23) | 325.0/138.9 (37) | 0.005 | 75.09 | 87.95 | 9.03 | 1.52 | 16.90 | 10.64 | 29 |
| Azinphos-ethyl | 346.0/132.1 (23) | 346.0(160.1 (15) | 0.006 | 91.16 | 112.74 | 13.35 | 2.59 | 11.57 | 15.00 | 22 |
| Azinphos-methyl | 318.0/132.0 (21) | 318.0/160.0 (13) | 0.005 | 83.68 | 98.02 | 9.25 | 3.31 | 14.62 | 13.91 | 22 |
| Azoxystrobin | 404.1/372.0 (21) | 404.1/344.1 (35) | 0.003 | 77.79 | 94.19 | 11.38 | 3.58 | 19.81 | 12.38 | 18 |
| Benalaxyl | 326.0/148.0 (27) | 326.0/208.0 (21) | 0.002 | 80.72 | 93.30 | 10.60 | 2.86 | 13.62 | 7.32 | 18 |
| Benazolin | 243.9/220.9 (32) | 243.9/170.0 (23) | 0.006 | 94.52 | 108.33 | 13.96 | 8.37 | 15.09 | 13.80 | 34 |
| Bendiocarb | 224.1/167.2 (13) | 224.1/108.9 (21) | 0.005 | 103.96 | 94.64 | 8.16 | 5.42 | 12.58 | 10.70 | 33 |
| Benodanil | 324.0/231.0 (13) | 324.0/120.0 (25) | 0.009 | 80.32 | 90.59 | 6.50 | 2.98 | 10.98 | 3.24 | 34 |
| Benoxacor | 260.1/149.1 (25) | 260.1/134.1 (41) | 0.003 | 84.62 | 97.41 | 10.66 | 4.25 | 10.72 | 9.67 | 20 |
| Bensulfuron-methyl | 411.1/148.9 (27) | 411.1/119.0 (51) | 0.005 | 81.60 | 88.98 | 5.21 | 2.21 | 9.00 | 8.03 | 33 |
| Bentazone | 239.0/131.9 (38) | 239.0/196.9 (28) | 0.004 | 79.39 | 76.91 | 6.58 | 2.67 | 13.24 | 9.19 | 22 |
| Bitertanol | 338.0/269.0 (15) | 338.0/70.0 (25) | 0.008 | 76.24 | 87.47 | 5.61 | 2.41 | 8.18 | 5.37 | 24 |
| Bixafen | 411.9/90.9 (58) | 411.9/279.8 (34) | 0.010 | 94.29 | 101.10 | 11.54 | 2.94 | 10.69 | 14.49 | 40 |
| Boscalid | 343.0/307.0 (27) | 343.0/140.0 (27) | 0.007 | 79.38 | 87.97 | 9.60 | 2.32 | 9.18 | 6.82 | 36 |

| | | | | | | | | | | |
|---|---|---|---|---|---|---|---|---|---|---|
| Bromacil | 261.0/205.0 (33) | 261.0/188.0 (36) | 0.005 | 77.82 | 72.72 | 6.98 | 3.50 | 12.91 | 14.31 | 32 |
| Bromoxynil | 275.7/78.6 (40) | 275.7/80.6 (42) | 0.006 | 78.26 | 82.12 | 10.29 | 4.21 | 19.96 | 7.22 | 22 |
| Bromuconazole | 378.0/159.0 (37) | 378.0/70.0 (35) | 0.007 | 103.41 | 100.84 | 4.99 | 17.98 | 18.32 | 8.87 | 21 |
| Bupirimate | 317.2/166.0 (35) | 317.2/108.0 (37) | 0.002 | 79.51 | 95.24 | 8.14 | 2.58 | 10.61 | 11.58 | 18 |
| Buprofezin | 306.2/201.0 (19) | 306.2/115.9 (23) | 0.002 | 79.37 | 90.65 | 10.04 | 2.96 | 10.21 | 4.49 | 18 |
| Butachlor | 312.2/238.1 (31) | 312.2/162.1 (35) | 0.003 | 87.48 | 102.14 | 9.13 | 3.53 | 11.59 | 3.80 | 15 |
| Butafenacil | 492.2/331.0 (31) | 492.2/180.2 (63) | 0.007 | 91.50 | 92.39 | 14.21 | 7.29 | 16.37 | 10.05 | 29 |
| Butamifos | 350.0/333.1 (15) | 350.0/152.0 (31) | 0.003 | 100.76 | 114.46 | 9.89 | 2.99 | 12.40 | 4.19 | 22 |
| Buturon | 237.2/84.2 (23) | 237.2/126.1 (41) | 0.003 | 87.86 | 100.84 | 9.15 | 2.74 | 11.33 | 11.17 | 18 |
| Butylate | 218.1/57.1 (29) | 218.1/156.2 (15) | 0.005 | 80.93 | 97.32 | 10.67 | 5.04 | 12.47 | 3.92 | 17 |
| Cadusafos | 271.1/158.9 (21) | 271.1/214.9 (13) | 0.002 | 87.71 | 99.08 | 10.86 | 2.89 | 10.75 | 8.08 | 19 |
| Carbaryl | 202.1/145.0 (15) | 202.1/127.0 (41) | 0.003 | 83.13 | 92.87 | 10.09 | 2.76 | 8.94 | 10.64 | 21 |
| Carbendazim | 192.1/160.0 (29) | 192.1/132.0 (45) | 0.006 | 98.35 | 85.12 | 18.20 | 6.94 | 17.03 | 14.56 | 28 |
| Carbofuran | 222.1/165.0 (17) | 222.1/123.1 (31) | 0.005 | 88.24 | 102.07 | 9.04 | 5.95 | 8.41 | 11.12 | 27 |
| Carboxin | 236.0/143.0 (21) | 236.0/87.0 (33) | 0.003 | 77.98 | 90.70 | 10.69 | 4.11 | 10.58 | 8.67 | 23 |
| Chlorbromuron | 295.1/225.9 (27) | 295.1/182.0 (23) | 0.007 | 86.75 | 91.11 | 3.41 | 3.30 | 8.45 | 5.04 | 33 |
| Chlorfluazuron | 539.9/383.0 (47) | 539.9/158.0 (47) | 0.006 | 78.81 | 77.68 | 6.91 | 7.01 | 17.37 | 12.49 | 26 |
| Chloridazon | 222.0/92.0 (35) | 222.0/104.0 (31) | 0.003 | 78.93 | 89.96 | 10.85 | 3.67 | 11.24 | 8.12 | 16 |
| Chloroxuron | 291.0/72.0 (41) | 291.0/218.1 (35) | 0.005 | 82.93 | 95.38 | 9.86 | 3.19 | 13.42 | 10.55 | 27 |
| Chlorpyrifos | 350.0/96.9 (49) | 350.0/197.9 (27) | 0.004 | 80.82 | 91.23 | 8.88 | 4.64 | 10.09 | 4.24 | 23 |
| Chlorsulfuron | 358.0/141.0 (23) | 358.0/167.0 (25) | 0.002 | 81.53 | 81.20 | 7.23 | 4.27 | 11.43 | 9.69 | 25 |
| Cinosulfuron | 414.1/183.1 (21) | 414.1/157.0 (33) | 0.004 | 78.81 | 82.41 | 13.48 | 4.11 | 12.64 | 13.89 | 25 |
| Clethodim | 360.0/164.0 (25) | 360.0/268.0 (17) | 0.003 | 83.19 | 92.64 | 10.67 | 8.25 | 18.08 | 13.24 | 20 |
| Clodinafop-propargyl | 350.1/266.0 (23) | 350.1/91.2 (43) | 0.004 | 98.95 | 110.93 | 9.12 | 5.03 | 10.61 | 16.75 | 19 |
| Clofentezine | 303.1/138.1 (21) | 303.1/102.2 (61) | 0.010 | 101.05 | 94.79 | 8.80 | 5.69 | 15.44 | 17.59 | 25 |
| Clomazone | 240.0/125.0 (27) | 240.0/89.0 (65) | 0.002 | 92.92 | 107.77 | 10.47 | 3.75 | 11.41 | 14.78 | 17 |
| Clopyralid | 191.9/146.0 (29) | 191.9/110.1 (47) | 0.008 | 91.50 | 110.12 | 10.13 | 4.00 | 11.89 | 10.28 | 31 |
| Clothianidin | 250.0/169.1 (17) | 250.0/132.0 (19) | 0.009 | 76.65 | 89.65 | 8.83 | 3.79 | 8.95 | 6.55 | 27 |
| Crimidine | 172.2/136.1 (29) | 172.2/107.1 (37) | 0.003 | 83.11 | 87.33 | 9.56 | 2.44 | 9.93 | 4.94 | 21 |
| Crotoxyphos | 315.1/211.0 (24) | 315.1/193.0 (27) | 0.004 | 80.50 | 96.20 | 7.88 | 2.82 | 9.58 | 8.25 | 28 |
| Cyanazine | 241.1/214.1 (25) | 241.1/216.1 (25) | 0.003 | 80.44 | 83.60 | 7.79 | 2.72 | 10.15 | 5.48 | 18 |
| Cyazofamid | 325.0/108.0 (19) | 325.0/261.0 (15) | 0.005 | 95.56 | 105.30 | 8.77 | 3.98 | 10.60 | 13.18 | 20 |
| Cycloate | 216.2/154.2 (17) | 216.2/134.2 (19) | 0.002 | 85.24 | 98.52 | 10.58 | 3.09 | 10.47 | 3.05 | 17 |
| Cyclohexamide | 282.2/264.0 (15) | 282.2/246.1 (21) | 0.008 | 80.72 | 82.14 | 9.43 | 6.56 | 13.93 | 6.57 | 44 |
| Cycloxydim | 326.2/280.1 (21) | 326.2/252.1 (33) | 0.002 | 93.49 | 104.69 | 10.44 | 1.94 | 10.71 | 4.05 | 18 |

| | | | | | | | | | | |
|---|---|---|---|---|---|---|---|---|---|---|
| Cycluron | 199.2/111.1 (35) | 199.2/89.1 (21) | 0.004 | 83.63 | 96.02 | 9.50 | 3.90 | 17.11 | 13.80 | 18 |
| Cyflufenamid | 413.2/295.0 (23) | 413.2/141.0 (33) | 0.003 | 96.42 | 112.44 | 10.76 | 1.72 | 11.23 | 7.09 | 20 |
| Cyhalofop-butyl | 375.1/256.1 (21) | 375.1/358.1 (11) | 0.005 | 90.36 | 103.94 | 13.96 | 12.78 | 13.62 | 12.87 | 24 |
| Cymoxanil | 199.0/127.9 (13) | 199.0/111.0 (18) | 0.007 | 82.67 | 84.65 | 8.66 | 3.80 | 14.33 | 8.24 | 36 |
| Cyproconazole | 292.0/70.0 (49) | 292.0/125.1 (37) | 0.008 | 81.08 | 91.88 | 8.86 | 4.77 | 8.73 | 12.71 | 28 |
| Cyprodinil | 226.1/93.1 (49) | 226.1/77.0 (65) | 0.003 | 80.87 | 95.16 | 9.46 | 2.53 | 9.05 | 13.44 | 17 |
| Daimuron | 269.2/151.1 (15) | 269.2/134.1 (19) | 0.003 | 77.10 | 92.41 | 9.83 | 3.20 | 14.79 | 12.50 | 18 |
| Demeton-s (disulfoton oxon) | 259.1/89.1 (6) | 259.1/61.0 (16) | 0.005 | 90.60 | 112.33 | 12.00 | 3.38 | 12.77 | 11.82 | 22 |
| Demeton-s-methyl-sulfone | 263.0/168.9 (23) | 263.0/120.8 (23) | 0.008 | 77.36 | 83.69 | 9.51 | 2.82 | 12.01 | 13.73 | 30 |
| Demeton-s-methyl-sulfoxide | 247.1/169.0 (17) | 247.1/125.0 (19) | 0.008 | 77.24 | 82.23 | 8.71 | 5.45 | 14.06 | 13.20 | 29 |
| Desmedipham | 318.0/136.0 (33) | 318.0/182.0 (19) | 0.002 | 77.13 | 93.18 | 9.07 | 3.71 | 10.67 | 12.27 | 16 |
| Desmetryn | 214.1/172.1 (5) | 214.1/82,0 (7) | 0.002 | 82.21 | 90.24 | 9.39 | 2.76 | 12.01 | 8.77 | 17 |
| Diafenthiuron | 385.2/329.2 (27) | 385.2/278.1 (43) | 0.005 | 84.57 | 99.32 | 13.40 | 8.08 | 12.35 | 8.83 | 30 |
| Diazinon | 305.1/169.0 (31) | 305.1/97.0 (47) | 0.003 | 82.93 | 98.92 | 11.63 | 3.00 | 11.42 | 4.25 | 19 |
| Diazinon-oxon | 289.1/261.1 (2) | 289.1/233.1 (4) | 0.002 | 86.02 | 86.62 | 8.40 | 3.69 | 12.75 | 8.73 | 20 |
| Dichlormid | 208,0/140.0 (27) | 208,0/98.1 (31) | 0.004 | 86.73 | 91.96 | 6.60 | 2.84 | 11.65 | 6.28 | 19 |
| Dichlorprop | 233.0/161.0 (5) | 233/125.0 (5) | 0.005 | 88.74 | 75.06 | 5.68 | 3.93 | 11.73 | 11.66 | 28 |
| Dichlorvos | 221.0/108.9 (25) | 221.0/127.0 (25) | 0.007 | 81.52 | 94.70 | 12.51 | 5.67 | 12.18 | 10.68 | 38 |
| Diclobutrazol | 328.1/199.0 (21) | 328.1/159.0 (26) | 0.006 | 78.60 | 92.75 | 13.63 | 3.63 | 13.56 | 12.08 | 24 |
| Dicrotophos | 238.0/112.0 (10) | 238.0/193.0 (10) | 0.006 | 81.78 | 85.67 | 10.06 | 8.69 | 11.45 | 7.93 | 23 |
| Diethofencarb | 268.1/226.1 (13) | 268.1/124.1 (41) | 0.004 | 79.25 | 90.17 | 10.11 | 3.27 | 16.97 | 8.86 | 18 |
| Difenoconazole | 406.0/251.0 (37) | 406.0/188.0 (37) | 0.005 | 89.01 | 87.96 | 8.01 | 2.93 | 11.12 | 3.31 | 23 |
| Difenoxuron | 287.1/214.1 (5) | 287.1/214.1 (5) | 0.002 | 88.44 | 96.40 | 8.48 | 2.92 | 17.26 | 11.51 | 17 |
| Diflubenzuron | 310.9/158.0 (6) | 310.9/141.0 (15) | 0.006 | 93.18 | 97.29 | 11.48 | 5.98 | 13.57 | 13.04 | 36 |
| Dimefuron | 339.1/295.1 (7) | 339.1/256.0 (18) | 0.003 | 87.45 | 93.23 | 10.34 | 4.03 | 12.94 | 9.75 | 18 |
| Dimethachlor | 256.4/224.3 (8) | 256.4/148.3 (24) | 0.003 | 81.69 | 95.65 | 9.82 | 3.01 | 10.07 | 14.38 | 18 |
| Dimethenamid | 276.1/244.1 (7) | 276.1/168.2 (21) | 0.003 | 94.27 | 93.11 | 8.25 | 4.37 | 13.42 | 11.37 | 16 |
| Dimethoate | 230.0/198.9 (3) | 230.0/125 (17) | 0.003 | 85.94 | 92.18 | 8.09 | 3.62 | 10.89 | 6.91 | 18 |
| Dimethomorph | 388.2/301 (17) | 388.2/165 (31) | 0.003 | 83.16 | 87.58 | 8.38 | 2.48 | 18.90 | 11.18 | 22 |
| Dimetilan | 241.0/196.1 (4) | 241.0/72.0 (14) | 0.003 | 80.50 | 83.61 | 7.69 | 3.07 | 10.90 | 5.85 | 18 |
| Dimoxystrobin | 327.1/205.1 (12) | 327.1/116.1 (23) | 0.004 | 80.38 | 91.17 | 9.07 | 3.78 | 14.76 | 7.15 | 19 |
| Diniconazole | 326.2/70.2 (25) | 326.2/43.2 (47) | 0.006 | 85.23 | 91.78 | 9.76 | 3.04 | 8.29 | 5.50 | 19 |
| Dinitramine | 323.0/289.0 (14) | 323.0/261.0 (12) | 0.007 | 97.65 | 86.69 | 9.68 | 4.38 | 19.20 | 3.92 | 30 |

| | | | | | | | | | | |
|---|---|---|---|---|---|---|---|---|---|---|
| Dinocap | 382.1/86.0 (2) | 382.1/69 (4) | 0.003 | 76.40 | 85.68 | 6.48 | 5.41 | 19.31 | 16.48 | 23 |
| Dinotefuran | 203.1/157.1 (21) | 203.1/129.1 (21) | 0.009 | 76.64 | 94.83 | 6.45 | 4.21 | 14.99 | 11.68 | 32 |
| Dioxacarb | 224.0/167.1 (2) | 224.0/123.0 (12) | 0.003 | 81.31 | 93.92 | 9.66 | 2.35 | 9.90 | 5.98 | 19 |
| Diphenamid | 240.3/167.1 (19) | 240.3/134.2 (17) | 0.002 | 84.43 | 89.64 | 11.31 | 3.87 | 14.78 | 9.96 | 18 |
| Dipropetryn | 256.2/144.1 (21) | 256.2/172.1 (21) | 0.002 | 92.35 | 91.05 | 8.13 | 3.77 | 14.23 | 11.69 | 17 |
| Disulfoton sulfone | 306.9/171.0 (8) | 206.9/153.1 (8) | 0.008 | 83.70 | 86.68 | 7.15 | 3.67 | 10.82 | 7.54 | 30 |
| Disulfoton sulfoxide | 290.9/184.9 (8) | 290.9/157 (20) | 0.009 | 84.00 | 88.47 | 11.26 | 3.84 | 10.27 | 5.29 | 35 |
| Dithianon | 295.9/263.8 (12) | 295.9/237.9 (12) | 0.007 | 99.98 | 96.45 | 8.00 | 7.11 | 19.98 | 16.00 | 41 |
| Dithiopyr | 402.1/382.1 (3) | 402.1/360.0 (3) | 0.004 | 85.14 | 81.95 | 7.63 | 2.57 | 13.74 | 3.88 | 20 |
| Diuron | 233.1/72.2 (17) | 233.1/46.3 (13) | 0.003 | 93.53 | 104.86 | 10.38 | 2.64 | 10.17 | 12.96 | 17 |
| Dodine | 228.2/71.1 (20) | 228.2/57.1 (22) | 0.002 | 91.16 | 92.16 | 7.14 | 3.50 | 13.06 | 10.89 | 25 |
| Edifenphos | 311.0/283.0 (12) | 311.0/220.0 (16) | 0.003 | 88.45 | 100.16 | 8.73 | 1.88 | 8.59 | 13.12 | 19 |
| Epoxiconazole | 330.1/121.1 (17) | 330.1/100.9 (40) | 0.003 | 94.56 | 100.10 | 8.36 | 2.80 | 12.69 | 13.79 | 22 |
| Eprinomectin | 914.5/186.1 (23) | 914.5/154.1 (23) | 0.009 | 79.86 | 89.30 | 14.20 | 5.26 | 19.13 | 8.39 | 45 |
| Esprocarb | 266.2/196.1 (6) | 266.2/142.1 (12) | 0.002 | 91.00 | 104.09 | 10.24 | 2.87 | 10.40 | 4.10 | 17 |
| Etaconazol | 330.0/161 (12) | 330.0/159 (12) | 0.003 | 80.90 | 93.04 | 10.54 | 4.29 | 14.03 | 12.62 | 20 |
| Ethametsulfuron-methyl | 411.0/196.0 (12) | 411.0/168.0 (34) | 0.003 | 91.73 | 89.10 | 11.28 | 3.10 | 14.62 | 8.37 | 24 |
| Ethidimuron | 265.0/208.0 (11) | 265.0/162.0 (15) | 0.003 | 87.85 | 91.08 | 7.52 | 3.20 | 12.16 | 5.07 | 20 |
| Ethiofencarb | 226.1/164.1 (3) | 226.1/107.1 (9) | 0.003 | 80.67 | 92.99 | 9.05 | 3.92 | 10.03 | 8.95 | 21 |
| Ethiofencarb sulfone | 258.1/201.1 (10) | 258.1/107.1 (20) | 0.002 | 84.86 | 82.93 | 7.23 | 3.36 | 10.94 | 8.76 | 17 |
| Ethiofencarb sulfoxide | 242.1/185.1 (5) | 242.1/107.1 (15) | 0.007 | 76.58 | 86.33 | 4.95 | 7.65 | 17.53 | 12.72 | 27 |
| Ethiolate | 162.1/134.1 (11) | 162.1/100.1 (14) | 0.003 | 81.16 | 93.68 | 9.69 | 4.47 | 11.51 | 5.59 | 19 |
| Ethion | 385.0/199.0 (3) | 385.0/143.0 (21) | 0.003 | 91.56 | 104.77 | 9.75 | 3.52 | 11.77 | 4.64 | 18 |
| Ethirimol | 210.1/140.2 (21) | 210.1/98.0 (27) | 0.002 | 87.21 | 87.54 | 7.39 | 3.38 | 8.55 | 10.72 | 20 |
| Ethofumesate | 287.1/259.1 (3) | 287.1/121.1 (11) | 0.006 | 87.19 | 96.35 | 12.75 | 3.31 | 15.18 | 14.11 | 24 |
| Ethoprophos | 243.0/172.9 (10) | 243.0/130.9 (20) | 0.003 | 91.30 | 105.86 | 9.41 | 4.11 | 12.87 | 16.52 | 19 |
| Etofenprox | 394.1/359.1 (2) | 394.1/177.0 (6) | 0.005 | 90.32 | 90.85 | 5.04 | 4.20 | 15.72 | 5.78 | 21 |
| Etoxazole | 360.0/141.0 (15) | 360.0/113.0 (23) | 0.003 | 84.86 | 83.36 | 8.46 | 3.27 | 11.14 | 5.85 | 18 |
| Famoxadone | 392.1/331.1 (2) | 392.1/238.1 (10) | 0.009 | 95.68 | 107.56 | 13.43 | 6.20 | 13.97 | 4.94 | 30 |
| Fenamidone | 312.1/236.1 (9) | 312.1/92.1 (20) | 0.003 | 84.23 | 86.88 | 9.08 | 4.56 | 14.07 | 7.93 | 19 |
| Fenamiphos | 304.1/234 (11) | 304.1/217.0 (19) | 0.002 | 80.53 | 94.35 | 11.80 | 3.23 | 11.16 | 10.10 | 21 |
| Fenamiphos-sulfone | 233.1/125.0 (20) | 233.1/161.0 (20) | 0.003 | 82.63 | 93.54 | 8.23 | 3.27 | 10.92 | 5.14 | 24 |
| Fenamiphos-sulfoxide | 262.1/220.1 (18) | 262.1/131.1 (31) | 0.003 | 83.25 | 92.20 | 10.40 | 3.02 | 11.73 | 10.28 | 17 |
| Fenazaquin | 307.2/161.1 (11) | 307.2/125.0 (31) | 0.003 | 81.73 | 88.15 | 9.85 | 1.17 | 13.55 | 8.88 | 18 |
| Fenbuconazole | 337.1/125.0 (31) | 337.1/70.1 (17) | 0.007 | 95.24 | 100.17 | 9.82 | 4.44 | 13.25 | 9.70 | 19 |

| | | | | | | | | | | |
|---|---|---|---|---|---|---|---|---|---|---|
| Fenhexamid | 302.0/97.0 (20) | 302.0/55.0 (35) | 0.009 | 90.10 | 93.79 | 4.99 | 3.86 | 9.39 | 7.39 | 31 |
| Fenobucarb | 208.1/152.1 (5) | 208.1/95.1 (10) | 0.003 | 82.00 | 97.21 | 8.66 | 3.01 | 8.82 | 12.41 | 18 |
| Fenothiocarb | 254.0/72.0 (14) | 254.0/160.0 (8) | 0.002 | 101.59 | 114.73 | 5.54 | 2.56 | 11.62 | 13.34 | 17 |
| Fenoxanil | 329.1/302.1 (3) | 329.1/189.1 (6) | 0.007 | 93.79 | 102.45 | 10.42 | 3.67 | 12.64 | 15.79 | 23 |
| Fenoxaprop-ethyl | 362.1/288.0 (15) | 362.1/244.1 (12) | 0.002 | 86.95 | 101.37 | 10.34 | 2.93 | 10.81 | 4.82 | 17 |
| Fenoxycarb | 302.1/287.9 (20) | 302.1/88.0 (11) | 0.004 | 98.56 | 104.81 | 10.64 | 3.63 | 15.02 | 15.31 | 26 |
| Fenpropathrin | 350.1/97.0 (34) | 350.1/125.0 (14) | 0.009 | 99.58 | 92.06 | 12.29 | 2.97 | 16.90 | 9.50 | 26 |
| Fenpropidin | 274.2/147.1 (30) | 274.2/117.0 (50) | 0.003 | 83.61 | 87.04 | 7.01 | 4.87 | 19.64 | 6.38 | 18 |
| Fenpropimorph | 304.4/147.1 (29) | 304.1/98.2 (29) | 0.003 | 82.50 | 84.91 | 8.12 | 3.32 | 11.38 | 9.43 | 20 |
| Fenpyrazamine | 332.1/304.1 (13) | 332.1/290.1 (13) | 0.003 | 76.87 | 89.42 | 8.44 | 3.43 | 15.09 | 8.28 | 21 |
| Fenpyroximate | 422.2/138.1 (32) | 422.2/366.1 (15) | 0.005 | 83.91 | 81.69 | 10.28 | 2.40 | 10.99 | 5.39 | 18 |
| Fensulfothion | 309.1/281.0 (9) | 309.1/253.0 (13) | 0.003 | 83.13 | 99.15 | 11.25 | 3.06 | 10.89 | 6.40 | 18 |
| Fenthion | 279.0/247.0 (5) | 279.0/169.0 (11) | 0.006 | 103.09 | 96.35 | 10.49 | 6.53 | 16.84 | 8.80 | 32 |
| Fenthion-oxon | 262.9/231.0 (12) | 262.9/215.9 (24) | 0.003 | 87.95 | 99.31 | 11.20 | 3.57 | 10.64 | 11.65 | 17 |
| Fenthion-oxonsulfone | 295.1/217.1 (14) | 295.1/104.1 (20) | 0.004 | 76.33 | 85.08 | 8.94 | 3.20 | 16.42 | 8.68 | 16 |
| Fenthion-oxonsulfoxide | 279.1/264.1 (14) | 279.1/104.1 (26) | 0.003 | 80.09 | 85.26 | 8.34 | 2.62 | 10.71 | 6.51 | 17 |
| Fenthion-sulfone | 311.0/279.9 (14) | 311.0/125.0 (28) | 0.005 | 92.56 | 94.84 | 9.75 | 6.14 | 14.31 | 5.01 | 22 |
| Fenthion-sulfoxide | 295.0/280.0 /14) | 295.0/109.0 (32) | 0.003 | 77.81 | 90.58 | 9.76 | 2.66 | 9.07 | 6.72 | 19 |
| Fenuron | 165.1/120.0 (3) | 165.1/95.0 (5) | 0.002 | 95.36 | 89.57 | 7.01 | 3.13 | 14.86 | 5.66 | 18 |
| Fipronil | 434.7/398.8 (4) | 434.7/329.8 (10) | 0.004 | 81.18 | 79.68 | 7.42 | 5.91 | 15.47 | 6.05 | 26 |
| Flamprop isopropyl | 364.1/105.0 (29) | 364.1/77.0 (73) | 0.002 | 91.83 | 104.91 | 8.33 | 2.04 | 11.08 | 12.25 | 19 |
| Flamprop-methyl | 336.1/105.0 (14) | 336.1/95.0 (20) | 0.003 | 90.18 | 104.95 | 6.71 | 2.53 | 10.86 | 15.09 | 20 |
| Florasulam | 360.4/192.1 (10) | 360.4/129.2 (24) | 0.009 | 85.55 | 91.79 | 12.23 | 4.33 | 9.83 | 8.56 | 26 |
| Fluazifop | 456.0/223.0 (15) | 456.0/344.0 (10) | 0.006 | 82.95 | 110.46 | 8.86 | 4.30 | 14.69 | 12.73 | 24 |
| Fluazifop-p-butyl | 384.2/328.1 (32) | 384.2/282.1 (20) | 0.002 | 81.77 | 97.30 | 10.69 | 2.66 | 11.71 | 3.71 | 18 |
| Fluazinam | 462.9/415.9 (13) | 462.9/397.9 (9) | 0.004 | 85.49 | 100.14 | 10.02 | 6.31 | 15.19 | 10.55 | 24 |
| Flubendiamide | 681.0/277.9 /24) | 681.0/253.9 (40) | 0.005 | 85.55 | 96.84 | 10.42 | 4.15 | 17.57 | 8.35 | 31 |
| Fludioxonil | 247.0/180.1 (22) | 247.0/126.0 (24) | 0.003 | 76.04 | 76.81 | 7.94 | 4.97 | 10.55 | 8.75 | 26 |
| Flufenoxuron | 489.1/141.1 (30) | 489.1/158.1 (15) | 0.007 | 82.60 | 83.02 | 14.94 | 4.96 | 15.05 | 10.67 | 25 |
| Flumetsulam | 326.0/262.1 (13) | 326.0/192.0 (23) | 0.002 | 99.35 | 93.64 | 6.71 | 4.41 | 14.10 | 4.74 | 16 |
| Fluometuron | 233.3/160.1 (24) | 233.3/72.4 (20) | 0.003 | 78.90 | 91.11 | 9.78 | 3.49 | 9.67 | 7.08 | 18 |
| Fluopyram | 397.0/207.8 (22) | 397.0/172.9 (32) | 0.009 | 89.02 | 95.96 | 12.53 | 4.43 | 12.62 | 9.91 | 34 |
| Fluoxastrobin | 459.4/427.4 (14) | 459.4/188.3 (36) | 0.002 | 85.92 | 91.18 | 6.80 | 2.54 | 10.31 | 11.54 | 19 |
| Fluridone | 330.1/310.1 (2) | 330.1/309.1 (2) | 0.003 | 92.33 | 91.73 | 7.93 | 2.87 | 16.22 | 9.85 | 17 |
| Flurochloridone | 311.9/291.9 (16) | 311.9/53.0 (30) | 0.008 | 87.89 | 100.08 | 11.46 | 6.06 | 15.45 | 12.43 | 44 |

| | | | | | | | | | | |
|---|---|---|---|---|---|---|---|---|---|---|
| Flutolanil | 324.0/262.0 (20) | 324.0/242.0 (20) | 0.003 | 78.29 | 92.58 | 9.25 | 2.94 | 9.52 | 6.15 | 22 |
| Flutriafol | 231.2/61.3 (27) | 231.2/89.2 (10) | 0.003 | 87.95 | 86.60 | 12.67 | 3.71 | 13.89 | 7.80 | 22 |
| Fluxapyroxad | 382.0/362.0 (10) | 382.0/342.0 (20) | 0.002 | 91.99 | 104.52 | 10.46 | 2.47 | 11.22 | 16.06 | 18 |
| Fomesafen | 456.1/344.0 (21) | 456.1/222.9 (45) | 0.006 | 76.31 | 73.68 | 8.20 | 3.04 | 13.86 | 16.60 | 28 |
| Forchlorfenuron | 248.2/155.1 (10) | 248.2/129.2 (14) | 0.005 | 76.74 | 74.42 | 11.92 | 2.51 | 11.05 | 12.53 | 24 |
| Formothion | 250.0/143.0 (6) | 250.0/258.0 (2) | 0.004 | 87.56 | 85.82 | 10.88 | 2.90 | 14.60 | 11.59 | 35 |
| Furalaxyl | 302.0/242.0 (15) | 302.0/95.0 (25) | 0.002 | 84.51 | 89.23 | 10.05 | 3.33 | 15.29 | 9.16 | 17 |
| Furathiocarb | 383.2/252.0 (7) | 383.2/195.0 (13) | 0.002 | 82.62 | 90.92 | 10.08 | 3.11 | 11.38 | 3.61 | 18 |
| Haloxyfop-etotyl | 434.0/316.0 (15) | 434.0/73.1 (13) | 0.003 | 83.74 | 98.02 | 11.67 | 3.64 | 11.67 | 4.89 | 19 |
| Haloxyfop-methyl | 376.0/316.1 (32) | 376.0/288.0 (25) | 0.003 | 90.67 | 107.37 | 11.36 | 3.17 | 11.38 | 4.52 | 17 |
| Heptenophos | 251.0/125.0 (15) | 251.0/109.0 (30) | 0.004 | 82.64 | 98.13 | 9.97 | 3.13 | 12.90 | 14.99 | 20 |
| Hexaconazole | 314.1/159.0 (31) | 314.1/70.1 (17) | 0.006 | 79.77 | 92.79 | 8.49 | 4.91 | 12.47 | 6.42 | 18 |
| Hexaflumuron | 461.0/158.0 (10) | 461.0/141.0 (20) | 0.006 | 88.65 | 75.70 | 8.76 | 4.58 | 14.07 | 13.81 | 26 |
| Hexazinone | 253.2/171.1 (11) | 253.2/71.2 (31) | 0.002 | 86.54 | 87.66 | 8.83 | 3.96 | 13.62 | 8.19 | 19 |
| Hexythiazox | 353.1/271.0 (6) | 353.1/228.0 (8) | 0.006 | 87.48 | 81.75 | 9.15 | 3.74 | 13.65 | 4.89 | 22 |
| Imazalil | 297.1/159.0 (19) | 297.1/41.2 (31) | 0.004 | 84.01 | 83.96 | 9.26 | 5.28 | 12.40 | 10.17 | 34 |
| Imazapyr | 262.0/217.0 (18) | 262.0/148.9 (26) | 0.009 | 99.47 | 84.40 | 5.68 | 3.39 | 15.26 | 11.38 | 28 |
| Imazaquin | 312.0/199.0 (29) | 312.0/128.0 (50) | 0.003 | 91.32 | 89.83 | 7.94 | 3.01 | 13.03 | 4.66 | 17 |
| Imibenconazole | 410.9/170.8 (18) | 410.9/125.0 (34) | 0.007 | 84.77 | 84.44 | 12.85 | 4.40 | 12.13 | 6.28 | 21 |
| Imidacloprid | 256.1/209.0 (10) | 256.1/175.0 (12) | 0.003 | 87.08 | 92.44 | 7.56 | 4.86 | 12.28 | 5.16 | 18 |
| Indanofan | 341.1/323.1 (0) | 341.1/295.1 (3) | 0.009 | 98.69 | 112.36 | 11.02 | 4.54 | 10.20 | 16.61 | 30 |
| Indoxacarb | 528.1/203.0 (36) | 528.1/150.0 (16) | 0.007 | 93.66 | 92.81 | 11.48 | 3.09 | 13.56 | 4.12 | 26 |
| Iodosulfuron-methyl sodium | 508.0/167.0 (17) | 508.0/141.0 (35) | 0.003 | 91.14 | 85.24 | 8.45 | 3.92 | 11.46 | 6.59 | 29 |
| Ioxynil | 369.8/215.0 (36) | 369.8/127.0 (30) | 0.008 | 87.43 | 84.31 | 8.53 | 6.25 | 15.40 | 5.19 | 23 |
| Ipconazole | 334.0/125.0 (48) | 334.0/70.0 (30) | 0.003 | 83.31 | 88.83 | 8.27 | 2.01 | 10.66 | 3.62 | 17 |
| Iprobenfos | 289.0/205.1 (4) | 289.0/91.0 (20) | 0.007 | 85.51 | 97.71 | 8.81 | 3.93 | 11.38 | 14.89 | 22 |
| Isocarbamid | 186.1/130.1 (7) | 186.1/87.1 (11) | 0.004 | 89.03 | 88.97 | 7.51 | 2.04 | 13.00 | 7.50 | 21 |
| Isoprocarb | 211.5/137.1 (13) | 211.5/95.0 (22) | 0.004 | 82.34 | 90.88 | 5.73 | 3.06 | 12.63 | 3.83 | 27 |
| Isopropalin | 310.2/276.2 (2) | 310.2/226.1 (4) | 0.005 | 86.59 | 87.58 | 9.75 | 3.04 | 10.85 | 7.61 | 21 |
| Isopyrazam | 360.2/340.2 (20) | 360.2/320.2 (24) | 0.003 | 80.90 | 95.94 | 9.54 | 2.73 | 8.17 | 3.94 | 25 |
| Isoxaben | 333.0/165.0 (17) | 333.0/107.0 (58) | 0.003 | 79.95 | 93.52 | 10.53 | 5.40 | 10.12 | 10.95 | 19 |
| Isoxathion | 314.1/170.0 (19) | 314.1/105.0 (23) | 0.004 | 84.44 | 100.74 | 9.73 | 3.32 | 9.42 | 4.57 | 19 |
| Kresoxim-methyl | 314.2/267.1 (3) | 314.2/222.1 (7) | 0.003 | 90.64 | 111.95 | 9.38 | 3.04 | 11.95 | 11.65 | 22 |
| Lactofen | 479.1/344.0 (0) | 479.1/223.0 (2) | 0.005 | 77.80 | 93.49 | 10.41 | 1.86 | 10.41 | 3.50 | 18 |
| Lenacil | 235.2/153.0 (11) | 235.2/136.0 (33) | 0.004 | 87.29 | 102.74 | 11.45 | 5.91 | 12.66 | 14.27 | 21 |

| | | | | | | | | | | |
|---|---|---|---|---|---|---|---|---|---|---|
| Linuron | 249.1/182.0 (11) | 249.1/160.0 (13) | 0.004 | 87.75 | 97.82 | 11.02 | 3.94 | 13.38 | 13.38 | 19 |
| Lufenuron | 510.9/158.0 (16) | 510.9/141.0 (44) | 0.006 | 98.23 | 94.29 | 6.28 | 4.65 | 10.29 | 13.17 | 29 |
| Malaoxon | 315.1/127 (10) | 315.1/99 (20) | 0.004 | 78.54 | 86.76 | 9.41 | 3.13 | 10.22 | 9.98 | 18 |
| Malathion | 330.9/285.0 (0) | 330.9/127.0 (4) | 0.003 | 86.57 | 100.59 | 10.36 | 3.58 | 10.45 | 14.33 | 20 |
| MCPA | 201.0/142.9 (10) | 201.0/140.9 (10) | 0.007 | 77.58 | 97.20 | 13.95 | 5.23 | 17.75 | 8.19 | 23 |
| Mecarbam | 330.1 /226.9 (5) | 330.1/198.9 (10) | 0.003 | 83.92 | 101.61 | 12.87 | 4.17 | 11.23 | 17.53 | 20 |
| Mefenacet | 299.1/192.0 (3) | 299.1/148.1 (3) | 0.006 | 90.37 | 92.14 | 6.97 | 2.44 | 11.70 | 9.68 | 22 |
| Mepanipyrim | 224.0/106.0 (26) | 224.0/77.1 (46) | 0.003 | 81.83 | 96.94 | 9.93 | 2.32 | 14.34 | 7.80 | 18 |
| Mepronil | 270.0/228.0 (14) | 270.0/119.0 (16) | 0.002 | 91.97 | 104.14 | 10.76 | 4.69 | 11.59 | 14.96 | 18 |
| Mesosulfuron-methyl | 502.1/346.9 (20) | 502.1/266.8 (34) | 0.006 | 82.01 | 86.02 | 13.03 | 5.18 | 16.62 | 17.68 | 30 |
| Metaflumizone | 505.0/302.0 (10) | 505.0/117.0 (48) | 0.010 | 81.45 | 84.48 | 14.82 | 3.52 | 13.74 | 7.30 | 27 |
| Metalaxyl-M | 280.2/220.2 (7) | 280.2/192.1 (13) | 0.002 | 97.29 | 94.92 | 8.02 | 4.01 | 16.14 | 8.33 | 19 |
| Metconazole | 321.0/125.0 (40) | 321.0/70.0 (22) | 0.003 | 81.34 | 92.79 | 10.23 | 3.11 | 12.48 | 4.84 | 21 |
| Methacrifos | 241.0/209.0 (5) | 241.0/124.9 (20) | 0.006 | 83.97 | 96.17 | 10.57 | 3.69 | 10.42 | 5.71 | 19 |
| Methamidophos | 142.0/124.9 (10) | 142.0/93.9 (10) | 0.007 | 85.27 | 81.57 | 11.65 | 4.26 | 14.99 | 8.51 | 27 |
| Methidathion | 302.9/145.0 (2) | 302.9/85.0 (10) | 0.004 | 84.40 | 98.04 | 9.41 | 4.54 | 11.20 | 14.53 | 19 |
| Methiocarb | 226.1/169.1 (5) | 226.1/121.1 (10) | 0.002 | 84.03 | 94.39 | 11.60 | 3.61 | 12.85 | 12.92 | 15 |
| Methiocarb-sulfone | 258.0/201.0 (14) | 258.0/122.0 (23) | 0.004 | 77.68 | 86.55 | 7.88 | 3.36 | 10.01 | 8.13 | 20 |
| Methiocarb-sulfoxide | 242.0/185.0 (14) | 242.0/122.1 (28) | 0.005 | 82.02 | 87.92 | 8.74 | 3.46 | 8.49 | 6.38 | 20 |
| Methomyl | 163.0/106.0 (5) | 163.0/88.0 (5) | 0.007 | 96.24 | 86.26 | 13.43 | 6.26 | 14.45 | 7.12 | 38 |
| Metobromuron | 259.0/169.9 (12) | 259.0/148.0 (8) | 0.006 | 82.22 | 93.03 | 12.85 | 4.64 | 10.35 | 5.79 | 37 |
| Metolachlor | 284.0/252.1 (8) | 284.0/176.1 (20) | 0.004 | 84.06 | 98.39 | 10.51 | 3.57 | 13.43 | 7.05 | 18 |
| Metosulam | 418.0/175.0 (25) | 418.0/140.0 (25) | 0.003 | 91.81 | 92.01 | 9.40 | 3.86 | 16.36 | 6.59 | 23 |
| Metoxuron | 229.0/156.0 (17) | 229.0/72.0 (17) | 0.003 | 82.02 | 88.26 | 7.67 | 2.58 | 8.69 | 6.79 | 23 |
| Metribuzin | 215.2/187.1 (13) | 215.2/84.1 (19) | 0.009 | 87.08 | 90.70 | 7.83 | 4.44 | 10.52 | 11.59 | 35 |
| Metsulfuron-methyl | 382.1/199.0 (19) | 382.1/167.0 (11) | 0.004 | 85.61 | 84.24 | 6.18 | 4.48 | 13.49 | 13.95 | 24 |
| Mevinphos | 225.1/193.0 (1) | 225.1/127.0 (11) | 0.003 | 79.39 | 85.59 | 8.09 | 3.78 | 9.77 | 8.44 | 19 |
| Molinate | 188.2/126.1 (9) | 188.2/55.2 (25) | 0.006 | 84.41 | 95.68 | 13.53 | 7.61 | 13.37 | 14.17 | 25 |
| Monocrotophos | 224.2/127.0 (8) | 224.2/58.0 (28) | 0.009 | 88.49 | 83.38 | 8.56 | 5.24 | 10.78 | 8.41 | 33 |
| Monolinuron | 215.0/148.0 (8) | 215.0/125.9 (12) | 0.002 | 81.39 | 93.19 | 10.46 | 3.70 | 9.98 | 6.63 | 18 |
| Monuron | 199.1/126.0 (20) | 199.1/72.0 (35) | 0.002 | 77.05 | 87.59 | 9.20 | 2.50 | 8.69 | 6.41 | 16 |
| Myclobutanil | 289.2/125.1 (27) | 289.2/70.2 (15) | 0.004 | 77.69 | 92.15 | 10.68 | 4.03 | 10.63 | 11.99 | 17 |
| Neburon | 275.1/88.2 (11) | 275.1/57.2 (19) | 0.007 | 89.58 | 101.67 | 8.06 | 3.59 | 11.05 | 15.67 | 29 |
| Nicosulfuron | 411.0/213.0 (10) | 411.0/182.0 (14) | 0.003 | 78.91 | 82.65 | 7.08 | 3.76 | 13.70 | 10.96 | 26 |
| Nitenpyram | 271.1/237.1 (15) | 271.1/224.0 (20) | 0.008 | 85.01 | 80.56 | 8.68 | 8.12 | 11.65 | 9.76 | 29 |

| | | | | | | | | | | |
|---|---|---|---|---|---|---|---|---|---|---|
| Nuarimol | 315.0/252.0 (16) | 315.0/81.0 (24) | 0.003 | 77.11 | 87.96 | 11.12 | 2.75 | 9.61 | 9.43 | 18 |
| Omethoate | 213.9/182.9 (4) | 213.9/125.0 (16) | 0.008 | 82.98 | 97.44 | 14.11 | 4.48 | 11.41 | 11.14 | 26 |
| Oxadixyl | 279.1/219.0 (2) | 279.1/102.0 (2) | 0.009 | 98.66 | 101.37 | 8.42 | 3.35 | 7.86 | 10.63 | 37 |
| Oxamyl | 237.0/90.0 (0) | 237.0/72.0 (10) | 0.008 | 102.23 | 106.34 | 6.91 | 5.95 | 17.60 | 9.02 | 35 |
| Penconazole | 284.1/159.0 (29) | 284.1/70.0 (13) | 0.004 | 90.96 | 103.64 | 10.54 | 4.23 | 10.43 | 16.50 | 18 |
| Pendimethalin | 282.2/212.0 (3) | 282.2/194.0 (3) | 0.006 | 80.46 | 89.66 | 6.21 | 4.78 | 6.87 | 5.94 | 20 |
| Phenmedipham | 301.0/168.0 (4) | 301.0/136.0 (18) | 0.003 | 81.07 | 87.22 | 12.88 | 4.36 | 14.57 | 10.42 | 18 |
| Phenthoate | 321.0/246.9 (6) | 321.0/163.0 (8) | 0.004 | 97.73 | 115.28 | 9.23 | 3.37 | 13.56 | 11.11 | 22 |
| Phosalone | 368.1/322.0 (3) | 368.1/182.0 (9) | 0.006 | 95.30 | 104.15 | 8.40 | 1.49 | 9.23 | 5.73 | 24 |
| Phosmet | 317.9/160.0 (5) | 317.9/133.0 (5) | 0.003 | 84.15 | 100.05 | 10.90 | 2.95 | 9.93 | 14.83 | 18 |
| Phosphamidon | 300.0/174.0 (8) | 300.0/127.0 (16) | 0.003 | 77.81 | 81.24 | 9.57 | 3.90 | 10.52 | 5.00 | 18 |
| Phoxim | 299.0/129.0 (2) | 299.0/97.0 (10) | 0.002 | 92.79 | 106.16 | 12.84 | 4.74 | 11.01 | 5.58 | 20 |
| Phthalide | 391.3/167.0 (5) | 391.3/149.0 (5) | 0.009 | 92.95 | 86.82 | 8.42 | 6.26 | 15.01 | 4.98 | 36 |
| Pirimicarb | 239.2/182.1 (11) | 239.2/72.1 (15) | 0.003 | 74.34 | 84.12 | 9.36 | 3.31 | 9.79 | 4.50 | 15 |
| Pirimiphos-ethyl | 334.2/198.1 (19) | 334.2/182.1 (19) | 0.003 | 81.40 | 94.96 | 11.70 | 3.99 | 10.95 | 4.47 | 18 |
| Pirimiphos-methyl | 306.2/164.1 (19) | 306.2/108.1 (31) | 0.003 | 82.13 | 96.35 | 9.45 | 3.92 | 8.67 | 5.75 | 19 |
| Primisulfuron | 469.0/254.0 (4) | 469.0/199.0 (6) | 0.008 | 108.93 | 78.09 | 7.40 | 5.38 | 11.26 | 16.40 | 23 |
| Prochloraz | 376.1/308.0 (5) | 376.1/265.9 (11) | 0.005 | 82.64 | 95.80 | 13.49 | 1.53 | 10.61 | 4.00 | 20 |
| Profenofos | 372.9/344.9 (10) | 372.9/302.9 (15) | 0.003 | 89.94 | 103.51 | 11.97 | 2.75 | 12.88 | 5.19 | 20 |
| Profoxydim | 466.1/280.1 (12) | 466.1/180.1 (24) | 0.004 | 83.44 | 82.06 | 13.72 | 3.47 | 13.48 | 8.06 | 23 |
| Prometryn | 242.5/200.3 (14) | 242.5/68.1 (44) | 0.003 | 77.12 | 87.22 | 9.09 | 4.63 | 8.56 | 9.31 | 16 |
| Propachlor | 212.2/170.1 (9) | 212.2/94.2 (25) | 0.002 | 87.87 | 100.70 | 10.09 | 3.78 | 10.30 | 9.17 | 17 |
| Propanil | 218.0/162.0 (15) | 218.0/127.0 (20) | 0.008 | 88.54 | 78.48 | 12.00 | 4.79 | 15.31 | 5.38 | 31 |
| Propaquizafop | 444.1/299.1 (20) | 444.1/100.1 (15) | 0.005 | 83.95 | 96.85 | 11.29 | 5.15 | 11.82 | 6.22 | 19 |
| Propargite | 368.1/231.1 (2) | 368.1/175.0 (8) | 0.004 | 81.48 | 95.38 | 9.45 | 3.13 | 8.75 | 4.36 | 20 |
| Propazine | 230.2/188.1 (13) | 230.2/146.1 (21) | 0.006 | 82.37 | 92.63 | 12.91 | 5.29 | 15.53 | 10.50 | 36 |
| Propham | 180.1/138.1 (1) | 180.1/120.1 (13) | 0.009 | 95.16 | 89.67 | 17.45 | 4.14 | 16.72 | 8.73 | 30 |
| Propiconazole | 342.2/159.0 (27) | 342.2/69.2 (17) | 0.004 | 85.40 | 91.24 | 9.72 | 3.62 | 10.53 | 6.76 | 21 |
| Propoxur | 210.2/111.1 (7) | 210.2/93.1 (21) | 0.008 | 95.20 | 109.73 | 17.12 | 3.82 | 17.54 | 7.18 | 34 |
| Propyzamide | 256.1/189.9 (9) | 256.1/172.9 (17) | 0.005 | 79.68 | 89.07 | 9.75 | 2.34 | 10.55 | 7.66 | 23 |
| Pymetrozine | 218.0/79.0 (16) | 218.0/79.0 (50) | 0.007 | 88.57 | 103.48 | 9.37 | 9.25 | 11.88 | 8.51 | 42 |
| Pyrazophos | 374.2/222.0 (17) | 374.2/194.0 (33) | 0.005 | 79.93 | 90.65 | 6.93 | 3.63 | 8.08 | 7.91 | 19 |
| Pyridaben | 365.2/309.1 (7) | 365.2/147.1 (23) | 0.004 | 82.61 | 87.04 | 9.55 | 2.97 | 11.09 | 5.26 | 21 |
| Pyridaphenthion | 314.2/205.0 (19) | 314.2/189.0 (17) | 0.006 | 80.91 | 95.56 | 9.36 | 2.68 | 11.11 | 14.13 | 21 |
| Pyridate | 379.2/351.1 (3) | 379.2/207.0 (11) | 0.005 | 79.07 | 87.20 | 8.33 | 2.50 | 16.92 | 5.72 | 18 |

| | | | | | | | | | | |
|---|---|---|---|---|---|---|---|---|---|---|
| Pyrifenox | 297.0/93.0 (15) | 297.0/93.0 (35) | 0.002 | 86.48 | 100.60 | 8.12 | 3.49 | 9.32 | 15.82 | 18 |
| Pyrimethanil | 200.3/107.0 (11) | 200.3/82.0 (23) | 0.002 | 83.06 | 92.45 | 8.38 | 3.20 | 8.96 | 8.89 | 17 |
| Pyriproxyfen | 322.2/185.0 (19) | 322.2/96.1 (11) | 0.009 | 81.82 | 87.62 | 10.37 | 3.43 | 10.50 | 4.38 | 18 |
| Quizalofop-p | 375.1/301.0 (15) | 375.1/299.0 (15) | 0.009 | 92.63 | 83.10 | 10.57 | 5.94 | 17.95 | 6.10 | 29 |
| Rimsulfuron | 432.1/182.0 (19) | 432.1/139.1 (60) | 0.004 | 82.58 | 82.43 | 9.60 | 4.44 | 11.45 | 7.91 | 46 |
| Sethoxydim | 328.2/282.2 (10) | 328.2/178.1 (15) | 0.003 | 78.72 | 89.66 | 10.89 | 4.37 | 11.47 | 5.60 | 20 |
| Simetryn | 214.1/186.1 (0) | 214.1/144.1 (2) | 0.002 | 75.44 | 89.28 | 10.29 | 2.80 | 10.33 | 8.72 | 17 |
| Spinosad A | 732.6/98.1 (59) | 732.6/142.0 (31) | 0.008 | 88.85 | 98.21 | 12.67 | 13.35 | 16.42 | 15.44 | 36 |
| Spinosad D | 746.5/98.1 (53) | 746.5/142.0 (31) | 0.004 | 92.30 | 108.58 | 17.33 | 10.85 | 18.49 | 12.82 | 17 |
| Spirodiclofen | 411.0/313.0 (11) | 411.0/71.0 (16) | 0.008 | 78.47 | 83.76 | 9.74 | 4.78 | 10.79 | 7.18 | 22 |
| Spiroxamine | 298.2/144.1 (14) | 298.2/100.0 (28) | 0.002 | 78.42 | 83.27 | 8.18 | 3.84 | 10.09 | 8.79 | 18 |
| Sulfluramid | 526.0/219.0 (7) | 526.0/169.0 (17) | 0.004 | 81.42 | 78.42 | 7.42 | 2.58 | 10.52 | 5.74 | 20 |
| Sulfosulfuron | 471.0/261.0 (19) | 471.0/211.0 (14) | 0.004 | 85.96 | 91.72 | 9.84 | 1.87 | 12.94 | 6.71 | 19 |
| Tebuconazole | 308.1/124.9 (36) | 308.1/70.0 (18) | 0.006 | 88.97 | 95.21 | 8.19 | 4.22 | 11.91 | 11.26 | 25 |
| Tebufenozide | 353.1/297.1 (2) | 353.1/133.0 (14) | 0.004 | 79.77 | 92.30 | 10.86 | 4.25 | 13.46 | 12.70 | 23 |
| Tebufenpyrad | 334.0/145.0 (28) | 334.0/117.0 (40) | 0.003 | 78.98 | 91.34 | 11.18 | 3.24 | 10.12 | 4.61 | 18 |
| Teflubenzuron | 379.0/359.0 (2) | 379.0/339.0 (2) | 0.005 | 82.85 | 82.05 | 9.81 | 4.14 | 18.98 | 14.03 | 24 |
| Terbutryn | 242.2/186.1 (13) | 242.2/91.1 (25) | 0.002 | 77.12 | 87.22 | 9.09 | 4.63 | 8.68 | 9.31 | 16 |
| Tetrachlorvinphos | 378.9/358,8 (10) | 378.9/96.2 (11) | 0.009 | 94.05 | 103.55 | 10.59 | 5.00 | 12.88 | 16.15 | 29 |
| Tetraconazole | 372.0/159.0 (35) | 372.0/70.0 (20) | 0.005 | 86.39 | 96.45 | 6.79 | 4.90 | 13.79 | 5.55 | 22 |
| Thiabendazole | 202.2/175.1 (25) | 202.2/131.1 (35) | 0.002 | 97.87 | 85.79 | 14.03 | 4.70 | 16.63 | 9.74 | 25 |
| Thiacloprid | 253.0/126.0 (16) | 253.0/90.0 (35) | 0.002 | 79.48 | 90.69 | 8.69 | 3.40 | 9.95 | 6.61 | 17 |
| Thiamethoxam | 292.1/211.1 (5) | 292.1/181.1 (19) | 0.008 | 79.33 | 85.15 | 7.91 | 6.60 | 9.09 | 10.21 | 31 |
| Thiobencarb | 258.1/125.1 (25) | 258.1/100.1 (5) | 0.004 | 90.97 | 103.28 | 9.81 | 3.40 | 10.67 | 5.29 | 20 |
| Thiodicarb | 355.1/108.0 (9) | 355.1/88.0 (9) | 0.006 | 82.63 | 89.49 | 6.06 | 4.27 | 10.94 | 5.97 | 37 |
| Tolylfluanid | 347.0/238.0 (5) | 347.0/137.0 (25) | 0.005 | 109.78 | 105.71 | 8.92 | 5.92 | 10.61 | 7.94 | 32 |
| Tralkoxydim | 330.3/284.1 (7) | 330.3/138.0 (17) | 0.005 | 76.92 | 83.00 | 8.24 | 3.99 | 8.27 | 5.37 | 18 |
| Triadimefon | 294.2/225.1 (7) | 294.2/197.1 (11) | 0.003 | 79.53 | 94.21 | 10.12 | 3.24 | 8.98 | 6.10 | 18 |
| Triadimenol | 296.1/99.0 (10) | 296.1/70.0 (5) | 0.007 | 86.18 | 92.98 | 8.72 | 4.65 | 14.17 | 9.21 | 33 |
| Triallate | 304.0/142.8 (22) | 304.0/86.0 (10) | 0.009 | 99.73 | 91.66 | 11.77 | 7.04 | 17.96 | 15.10 | 39 |
| Triasulfuron | 402.1/167.1 (25) | 402.1/140.8 (29) | 0.005 | 100.49 | 101.41 | 15.38 | 1.82 | 15.58 | 11.81 | 27 |
| Trichlorfon | 256.8/221.0 (4) | 256.8/109.0 (12) | 0.007 | 85.69 | 87.50 | 7.76 | 3.87 | 10.79 | 4.86 | 21 |
| Trifloxystrobin | 409.3/206.0 (9) | 409.3/186.0 (13) | 0.002 | 80.16 | 94.16 | 10.89 | 3.31 | 10.43 | 4.92 | 18 |
| Triflumizole | 346.2/278.0 (3) | 346.2/43.2 (21) | 0.002 | 81.08 | 91.59 | 9.31 | 3.16 | 9.82 | 4.19 | 18 |
| Triflumuron | 359.0/156.0 (5) | 359.0/139.0 (15) | 0.007 | 89.73 | 91.06 | 12.69 | 4.04 | 14.89 | 5.90 | 27 |

| | | | | | | | | | | |
|---|---|---|---|---|---|---|---|---|---|---|
| Triticonazole | 318.0/125.0 (30) | 318.0/70.0 (20) | 0.004 | 82.00 | 91.21 | 9.71 | 4.52 | 13.16 | 11.79 | 18 |
| Vamidothion | 288.1/146.1 (5) | 288.1/58.2 (43) | 0.006 | 78.98 | 84.96 | 9.34 | 3.25 | 11.20 | 8.52 | 32 |

**Table S2. MRM transitions and in-house validation data for 46 pesticides by GC-MS/MS**

| **Pesticide** | **MRM transitions, *m/z* (collision energy, eV)** | | **LOQ (mg $kg^{-1}$)** | **Recovery (%)** | | **Repeatability (%RSD, *n*=6)** | | **Within-laboratory reproducibility (%RSD, *n*=12)** | | ***U* (%)** |
|---|---|---|---|---|---|---|---|---|---|---|
| | quantification | qualification | | 0.01 mg $kg^{-1}$ | 0.05 mg $kg^{-1}$ | 0.01 mg $kg^{-1}$ | 0.05 mg $kg^{-1}$ | 0.01 mg $kg^{-1}$ | 0.05 $mgkg^{-1}$ | |
| Acetochlor | 132.1/117.1 (10) | 132.1/131.1 (10) | 0.010 | 85.76 | 107.43 | 11.60 | 9.43 | 12.27 | 8.25 | 33 |
| Alachlor | 160.1/131.7 (10) | 160.1/188.1 (8) | 0.010 | 81.57 | 88.33 | 5.45 | 5.67 | 11.89 | 12.13 | 29 |
| Aldrin | 91.1 /65.0 (15) | 91.1 /192.9 (30) | 0.010 | 77.37 | 88.95 | 5.94 | 4.63 | 16.50 | 11.73 | 32 |
| Atrazine | 226.3/184.2 (19) | 226.3/99.1 (20) | 0.010 | 77.59 | 83.38 | 4.08 | 6.60 | 15.65 | 14.30 | 34 |
| Benfluralin | 292.1./264.0 (15) | 292.1/160 (10) | 0.006 | 77.58 | 76.15 | 3.41 | 4.42 | 14.52 | 13.12 | 24 |
| Bifenthrin | 181.1/160.1 (25) | 181.1/166.1 (10) | 0.008 | 82.00 | 88.20 | 4.09 | 3.05 | 11.58 | 7.84 | 19 |
| Biphenyl | 153.1/152.1 (15) | 154.1/153.1 (15) | 0.010 | 106.14 | 98.99 | 3.35 | 10.37 | 6.78 | 8.37 | 37 |
| Butralin | 266/147.1 (24) | 266/174.1 (21) | 0.007 | 92.95 | 74.87 | 2.73 | 3.50 | 11.97 | 13.64 | 31 |
| Captan | 151.0/79.0 (20) | 151.0/122 (10) | 0.010 | 87.89 | 88.10 | 15.19 | 7.54 | 11.75 | 11.99 | 43 |
| Carbosulfan | 381.3/160.2 (9) | 381.3/118.2 (15) | 0.008 | 82.65 | 85.55 | 3.57 | 6.07 | 15.71 | 14.35 | 45 |
| Chlorothalonil | 265.9/230.8 (14) | 265.9/168.0 (22) | 0.009 | 80.17 | 73.77 | 7.41 | 3.02 | 17.17 | 16.71 | 37 |
| Chlorpropham | 213.8/171.8 (2) | 213.8/153.8 (12) | 0.009 | 92.63 | 82.49 | 2.12 | 2.85 | 15.41 | 12.14 | 37 |
| Chlorpyrifos-methyl | 321.9/289.9 (11) | 321.9/125.0 (17) | 0.009 | 83.62 | 72.28 | 6.30 | 2.30 | 15.45 | 16.83 | 36 |
| Cyanophos | 243.0/109.0 (12) | 243.0/125.0 (14) | 0.008 | 73.82 | 71.87 | 3.64 | 3.65 | 19.79 | 17.17 | 40 |
| Deltamethrin | 252.9/93.0 (20) | 252.9/171.9 (8) | 0.010 | 90.70 | 73.93 | 4.10 | 2.67 | 14.81 | 17.31 | 43 |
| Dichlofluanid | 331.1/224.0 (5) | 331.1/123.0 (25) | 0.007 | 114.42 | 101.63 | 2.43 | 6.74 | 18.91 | 7.74 | 47 |
| Dicofol | 250.0/139.0 (14) | 250.0/215.0 (8) | 0.008 | 81.80 | 89.73 | 3.36 | 3.62 | 11.65 | 8.07 | 28 |
| Dieldrin | 276.9/241.0 (8) | 276.9/170.0 (38) | 0.009 | 90.59 | 94.40 | 5.31 | 3.20 | 8.32 | 7.07 | 29 |
| Dinobuton | 211.0/163.0 (8) | 211/117.0 (18) | 0.010 | 104.57 | 92.01 | 7.33 | 8.25 | 16.38 | 7.33 | 37 |
| Disulfoton | 275.0/89.0 (5) | 275.0/61.0 (20) | 0.009 | 73.08 | 77.53 | 2.20 | 6.55 | 16.61 | 12.72 | 38 |
| Endrin | 262.9/193.0 (28) | 262.9/191.0 (30) | 0.009 | 72.45 | 83.49 | 2.51 | 3.57 | 14.89 | 10.54 | 31 |
| Esfenvalerate | 439.0/169.0 (10) | 439.0/167.0 (14) | 0.010 | 76.23 | 72.46 | 3.88 | 2.00 | 13.39 | 12.35 | 41 |
| Fenarimol | 330.8/267.9 (20) | 330.8/81.1 (32) | 0.009 | 81.98 | 85.97 | 2.25 | 2.44 | 12.48 | 10.09 | 31 |
| Fenvalerate | 225.1/119.1 (15) | 225.1/167.1 (15) | 0.009 | 77.16 | 72.33 | 3.51 | 2.26 | 13.11 | 12.92 | 37 |
| Flumetralin | 143.0/107.0 (18) | 143.0/117.0 (20) | 0.009 | 116.09 | 78.05 | 1.08 | 5.14 | 18.10 | 14.53 | 34 |
| Folpet | 259.9/130.0 (14) | 259.9/95.0 (20) | 0.008 | 84.79 | 84.20 | 3.44 | 9.02 | 12.54 | 17.84 | 37 |
| Fonofos | 247.0/137.0 (5) | 247.0/109 (15) | 0.010 | 75.98 | 80.02 | 5.39 | 5.98 | 14.17 | 10.68 | 34 |

| | | | | | | | | | | |
|---|---|---|---|---|---|---|---|---|---|---|
| Heptachlor | 271.8/236.9 (20) | 271.8/117.0 (32) | 0.008 | 74.16 | 85.47 | 2.93 | 4.33 | 14.04 | 12.43 | 27 |
| Iprodione | 330.0/287.9 (5) | 330.0/245.0 (12) | 0.009 | 87.48 | 73.88 | 7.78 | 1.97 | 17.71 | 17.17 | 32 |
| Metamitron | 203.2/104.0 (19) | 203.2/42.0 (29) | 0.010 | 99.30 | 84.87 | 7.86 | 10.05 | 16.81 | 15.97 | 30 |
| Nitrapyrin | 193.9/133.0 (16) | 193.9/157.9 (20) | 0.010 | 77.14 | 72.92 | 9.18 | 1.60 | 18.79 | 14.31 | 42 |
| Oxyfluorfen | 362.0/316.0 (10) | 362.0/237.1 (25) | 0.006 | 81.50 | 74.70 | 2.58 | 2.50 | 18.23 | 15.44 | 31 |
| Parathion-ethyl | 292.0/264.0 (5) | 292.0/236.0 (10) | 0.010 | 79.60 | 74.57 | 4.57 | 3.99 | 14.18 | 13.40 | 35 |
| Parathion-methyl | 264.0/231.9 (23) | 264.0/124.9 (27) | 0.010 | 104.46 | 73.87 | 3.70 | 2.20 | 12.89 | 14.62 | 38 |
| Permethrin. cis- | 183.1/168.1 (14) | 183.10/165.1 (10) | 0.009 | 78.93 | 84.66 | 2.96 | 2.92 | 14.31 | 8.54 | 22 |
| Permethrin. trans- | 183.1/168.1 (14) | 183.10/165.1 (10) | 0.010 | 76.16 | 76.98 | 3.21 | 3.02 | 14.68 | 11.74 | 28 |
| Phorate | 261.0/75.0 (10) | 261.0/47.0 (28) | 0.009 | 72.88 | 77.97 | 2.70 | 6.68 | 15.01 | 13.43 | 46 |
| Propamocarb | 189.1/102.1 (10) | 189.1/144.1 (10) | 0.009 | 76.48 | 72.40 | 4.34 | 2.06 | 13.52 | 16.10 | 39 |
| Prothiofos | 345.0/268.9 (5) | 345.0/240.9 (13) | 0.009 | 76.00 | 83.86 | 5.13 | 3.58 | 11.22 | 9.25 | 27 |
| Pyrimidifen | 384.1/282.0 (18) | 384.1/328.0 (14) | **0.011** | 71.77 | 72.30 | 1.03 | 2.59 | 19.62 | 16.09 | 26 |
| Simazine | 202.1/124.2 (13) | 202.1/104.0 (13) | 0.010 | 104.90 | 77.57 | 11.93 | 4.72 | 12.21 | 15.51 | 32 |
| Tau-fluvalinate | 250.1/55.0 (20) | 250.1/200.0 (20) | 0.010 | 76.27 | 72.18 | 3.88 | 2.46 | 11.09 | 17.02 | 40 |
| Terbufos | 288.9/103 (2) | 288.9/57.1 (18) | 0.008 | 73.00 | 78.31 | 1.80 | 3.79 | 15.71 | 12.78 | 31 |
| Tetramethrin | 332.0/286.0 (10) | 332.0/164.0 (29) | 0.010 | 77.15 | 83.48 | 3.73 | 3.06 | 13.91 | 11.65 | 33 |
| Trifluralin | 306.1/264.1 (8) | 306.1/206.1 (14) | 0.008 | 73.58 | 78.37 | 2.09 | 5.23 | 12.38 | 10.56 | 29 |
| Vinclozolin | 285.0/212.0 (12) | 285.0/178.0 (14) | 0.009 | 74.74 | 80.11 | 5.18 | 4.58 | 12.77 | 10.46 | 31 |

**Table S3.** The presence and quantification of pesticide residues in whole lemon fruits.

| Pesticide | Type of residue | EU MRL (mg kg$^{-1}$) | % of samples <LOQ | % of samples between LOQ-MRL | % of samples >MRL | Range (mg kg$^{-1}$) | |
|---|---|---|---|---|---|---|---|
| | | | | | | Min.-Max. | Mean |
| 2-phenilphenol | FU[a] | 10.0 | 99 | 1 | - | 0.552 | 0.552 |
| Acetamiprid | IN[b] | 0.9 | 96 | 4 | - | 0.051-0.128 | 0.090 |
| Azoxystrobin | FU | 15.0 | 98 | 2 | - | 0.040-0.044 | 0.042 |
| Buprofezin | IN | 0.01 | 91 | - | 9 | 0.023–0.076 | 0.050 |
| Chlorpyrifos* | IN/AC[c] | 0.01 | 99 | - | 1 | 0.073 | 0.073 |
| Chlorpyrifos-methyl* | IN/AC[c] | 0.01 | 83 | - | 17 | 0.013-0.098 | 0.056 |
| Difenoconazole | FU | 0.6 | 99 | 1 | - | 0.025 | 0.025 |
| Imazalil | FU | 5.0 | 95 | 5 | - | 0.419–1.172 | 0.796 |
| Malathion | FU | 2.0 | 93 | 7 | - | 0.100-0.482 | 0.291 |
| Metamitron | HB[d] | 0.01 | 90 | - | 10 | 0.027–0.118 | 0.073 |
| Pirimiphos-methyl | IN | 0.01 | 99 | - | 1 | 0.056 | 0.056 |
| Prochloraz | FU | 0.03 | 97 | - | 3 | 0.089–0.928 | 0.509 |
| Propiconazole* | FU | 0.01 | 99 | - | 1 | 0.040 | 0.040 |
| Pyrimethanil | FU | 8.0 | 95 | 5 | - | 0.033–0.548 | 0.290 |
| Pyriproxyfen | IN | 0.6 | 91 | 9 | - | 0,021-0,102 | 0.061 |
| Thiabendazole | FU | 7.0 | 98 | 2 | - | 0,104 – 0.111 | 0.108 |

[a]FU: fungicide. [b]IN: insecticide. [c]AC: acaricide. [d]HB: herbicide. *Not approved in the EU.
